# Comparison of chondrogenesis-related biological behaviors between human urine-derived stem cells and human bone marrow mesenchymal stem cells from the same individual

**DOI:** 10.1186/s13287-021-02370-1

**Published:** 2021-06-28

**Authors:** Jiachen Sun, Fei Xing, Min Zou, Min Gong, Lang Li, Zhou Xiang

**Affiliations:** 1 Department of Orthopedics, West China Hospital, Sichuan University, Chengdu, Sichuan 610041 People’s Republic of China; 2 Department of Orthopedics, NO. 1 People’s Hospital of Chengdu, Chengdu, Sichuan 610016 People’s Republic of China; 3 Department of Orthopedics, Hospital of Chengdu University of Traditional Chinese Medicine, Chengdu, Sichuan 610075 People’s Republic of China; 4 Department of Orthopaedics, Hospital of Chengdu Office of People’s Government of Tibetan Autonomous Region, Chengdu, Sichuan 610041 People’s Republic of China

**Keywords:** Human urine-derived stem cells, Human bone marrow mesenchymal stem cells, Stem cells, Acellular cartilage extracellular matrix, Cartilage repair, Hyaline cartilage regeneration

## Abstract

**Background:**

Stem cells are the main choice for seed cells in tissue engineering, but using most traditional stem cells requires invasive and complicated procedures. Human urine-derived stem cells (hUSCs) are an alternative stem cell source with the advantages of being isolated noninvasively and repetitively from the same individual. The aim of this study was to compare chondrogenesis-related biological behaviors between hUSCs and human bone marrow mesenchymal stem cells (hBMSCs) from the same individual.

**Methods:**

hUSCs and hBMSCs were isolated from six patients who underwent iliac bone grafting. Cell morphology, proliferation, colony-forming, migration, and multidifferentiation analyses were performed in vitro. Then, acellular cartilage extracellular matrix (ACM) scaffolds were fabricated for in vivo implantation. The comparisons of cell viability, morphology, proliferation, and chondrogenesis between hUSCs and hBMSCs cultured on scaffolds were performed before implantation. The scaffolds loaded with hUSCs or hBMSCs were implanted into a rabbit knee model to repair cartilage defects. Magnetic resonance imaging (MRI) and micro-computed tomography (μCT) Analyses, inflammation and toxicity assays, gross observation, and histological evaluation were performed to evaluate the cartilage repair effects.

**Results:**

In in vitro experiments, hUSCs had better capacity for proliferation, colony-forming, and migration compared to hBMSCs in the same passage, while hBMSCs had greater osteogenic, adipogenic, and chondrogenic abilities compared to hUSCs in the same passage. Both hUSCs and hBMSCs at passage 3 had the strongest potential for proliferation, colony-forming, and multilineage differentiation compared to cells in other passages. The ACM scaffolds loaded with hUSCs or hBMSCs both significantly promoted the repair of cartilage defects in the rabbit knee model at 12 weeks’ postimplantation, and the new tissue was mainly hyaline cartilage. However, there was no significant difference in cartilage repair effects between hUSCs and hBMSCs.

**Conclusions:**

In in vitro experiments, hUSCs presented better capacity for proliferation, while hBMSCs had greater chondrogenic ability. However, hUSCs and hBMSCs had similar cartilage repair effects in vivo. Results indicated that hUSCs can be a stem cell alternative for cartilage regeneration and provide a powerful platform for cartilage tissue engineering and clinical transformation.

**Graphical abstract:**

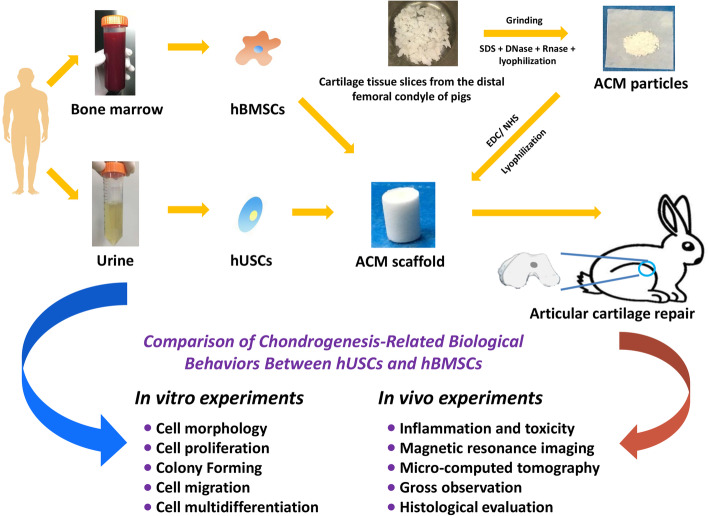

**Supplementary Information:**

The online version contains supplementary material available at 10.1186/s13287-021-02370-1.

## Introduction

With an increasing number of people exercising and an aging population, cases of articular cartilage injury are increasing yearly worldwide [1]. Articular cartilage lesions, accompanied by continuous degeneration of the surrounding area, often result in joint pain and dysfunction, seriously affecting patients’ life and work. Articular cartilage consists of hyaline cartilage and lacks blood supply, so an injury is difficult to heal [2]. Currently, clinical treatment options for severe articular cartilage injury involve chondroplasty, microfracture, mosaicplasty, and autologous chondrocyte implantation. However, these methods are complex and costly, have few donor sources, and cannot completely stimulate the production of hyaline cartilage. Therefore, to simulate the biological properties of natural articular cartilage and integrate repaired tissue with adjacent native tissue, tissue engineering is considered to be a promising strategy for cartilage regeneration [3–5].

Seed cells commonly used in cartilage tissue engineering include chondrocytes and stem cells from various sources [6]. Although autologous chondrocytes are the most ideal choice, their acquisition rate is low, the cost to obtain them is high, and there are some unfavorable factors after isolation, such as trauma, pain, infection, and even joint dysfunction [7]. These shortcomings limit the wide use of chondrocytes in clinical treatment. Therefore, all kinds of stem cells that can be cultured in vitro and have the ability to proliferate and differentiate are more often used as seed cells in cartilage tissue engineering [8]. However, traditional autologous stem cells, such as human bone marrow mesenchymal stem cells (hBMSCs), human adipose-derived stem cells (hADSCs), placenta decidua basalis-derived mesenchymal stem cells (hPDB-MSCs), and induced pluripotent stem cells (iPSCs), have some disadvantages, such as relatively few sources, invasive procedures necessary to obtain them, and tumorigenesis after implantation [9, 10]. Therefore, in recent years, seed cells are often excluded in many studies of tissue-engineered cartilage, and a variety of bioactive factors are used to promote the homing, proliferation, and differentiation of cartilage repair-related cells, combined with a complex design of scaffold materials, to simulate the biological environment of cartilage regeneration synergistically [11]. Because of the use of multiple bioactive factors and the complex preparation process of the scaffold, preparing many tissue-engineered cartilages is very complicated, and the material cost is very high, which affects the process of translating tissue engineering research to clinical applications.

Human urine-derived stem cells (hUSCs) are autologous stem cells with a low cost to isolate in a noninvasive procedure and have the ability to stably proliferate and differentiate. These stem cells are expected to solve challenges in cartilage tissue engineering research at the present stage and improve the possibility of clinical applications of stem cells and tissue engineering outcomes [12, 13]. hUSCs can be isolated from human urine with little ethical controversy, and they have been proved to differentiate into urinary system cells, osteoblasts, neuron-like cells, smooth muscle cells, and so on to promote related tissue regeneration. Some studies have reported that hUSCs showed lower osteogenic and chondrogenic differentiation rates compared to those of hBMSCs or hADSCs in vitro [14, 15]. However, many factors are involved in the repair of articular cartilage injury in vivo, and there is no comparative study on the repair of cartilage defects by hUSCs and other traditional stem cells [16]. Therefore, relevant in vivo experiments are needed to further compare the clinical application prospects of hUSCs and other traditional stem cells in the treatment of cartilage injury.

Scaffold material is another key element of cartilage tissue engineering that can provide structural and mechanical support as well as a microenvironment for cell growth and cartilage differentiation [17, 18]. The acellular cartilage extracellular matrix (ACM) and natural cartilage tissue have similar components, such as type II collagen (Col II) and glucosaminoglycans (GAGs), which can interact with seed cells and promote cell adhesion, proliferation, and differentiation [19, 20]. In addition, the ACM scaffold has good histocompatibility and can be remodeled instead of being metabolized and removed during new tissue formation, so it does not have immune rejection or degradation problems [21].

In this study, chondrogenesis-related biological behaviors between hUSCs and hBMSCs from the same individual were analyzed in vitro, and these two types of stem cells were loaded onto ACM scaffolds for comparing cartilage defect repair in vivo. The experimental results compare the effects of hUSCs and traditional stem cells on cartilage regeneration and provide a reference for applying hUSCs in the treatment of cartilage injury as well as a new direction for the clinical transformation of stem cells and tissue engineering outcomes.

## Materials and methods

The Research Ethics Board for both human samples and animal protocols was approved by the Ethics Committee of West China Hospital, Sichuan University, Chengdu, China. And the experimental protocol was also approved by the ethics committee. All the reagents were purchased from Sigma-Aldrich (St. Louis, MO, USA), cell culture media were supplied by HyClone (Logan, UT, USA), and cell induction media were supplied by Cyagen (Guangzhou, Guangdong, China) unless otherwise stated.

### Isolation and culture of hBMSCs and hUSCs

Human bone marrow and urine samples were obtained from six patients who underwent iliac bone grafting at the orthopedic department of the West China Hospital after providing written informed consent (Table 1). The body mass index of all patients ranged from 18 to 26 kg/m^2^. These patients did not have blood, urinary, metabolic, and other diseases. Bone marrow samples were collected from the patients’ iliac crest, and hBMSCs were isolated using the method outlined in a previous report [15]. Briefly, 1 ml of bone marrow aspirate was diluted with 2 mL of phosphate-buffered saline (PBS; pH 7.2) and 5 U/mL of heparin, and the mixture was gently loaded onto 3 mL of Lymphoprep™ (Solarbio, Beijing, China). Centrifugation was performed at 500×*g* for 40 min, and the interface layer, which included the desired mononuclear cells, was taken out and diluted with PBS. Then, the mixture was resuspended in 10 mL of growth medium (Dulbecco’s modified Eagle’s medium-high glucose [DMEM-HG, Gibco, Billings, MT, USA] containing 10% fetal bovine serum [FBS, Gibco] and 1% penicillin-streptomycin solution [Solarbio]) and seeded into T-75 flasks at a density of 2 × 10^5^ cells/cm^2^, followed by incubation with 5% CO_2_ at 95% humidity and 37°C. The culture medium was replaced every 48 h. When the hBMSCs reached 70–80% confluence, they were digested using 0.25% trypsin/ethylenediaminetetraacetic acid (EDTA) and passaged into new flasks at a ratio of 1:3. The 3rd, 5th, and 7th passage (P3, P5, and P7) hBMSCs were used in this study.

**Table 1:** Characterization of patients

Donor	Gender	Age (years)	Indication	Urine collection source	Bone marrow collection source
1	Male	42	Burst fracture of the fifth lumbar vertebra	Clean midstream urine	Left posterior superior iliac spine
2	Female	27	Delayed union of right tibial fracture	Urinary catheterization	Right anterior superior iliac spine
3	Female	39	Nonunion of left femoral fracture	Urinary catheterization	Left anterior superior iliac spine
4	Male	34	Delayed union of right ankle fracture	Clean midstream urine	Right anterior superior iliac spine
5	Male	39	Delayed union of right ankle fracture	Clean midstream urine	Left anterior superior iliac spine
6	Male	30	Comminuted fracture of the left calcaneus	Clean midstream urine	Left anterior superior iliac spine

Urine samples were collected from the same patients before surgery. Primary hUSCs were isolated using the method outlined in our previous report [22]. Briefly, 200 mL of sterile urine was collected from each person, with 1% penicillin-streptomycin solution added to each sample, and centrifuged at 400×*g* for 10 min. The cell pellet with 5 ml of supernatant was resuspended in 25 mL of PBS and centrifuged again at 400×*g* for 10 min followed by one further round of centrifuging. After the final centrifugation, the supernatant was discarded. The precipitated cells were resuspended in 5 mL of complete medium containing 50% keratinocyte serum-free medium (K-SFM, Gibco), 32.75% DMEM-HG, 11.25% Ham’s F12 (Hyclone), 5% FBS, 1% penicillin-streptomycin solution, and several supplements (5 ng/mL epidermal growth factor [EGF], 50 ng/mL bovine pituitary extract [BPE], 0.4 μg/mL hydrocortisone, 5 μg/mL transferrin, 5 ng/mL bovine insulin, 0.18 mmol/L adenine, and 2 nmol/L 3,3,5-triiodo-l-thyromine) and seeded into T-25 flasks. The cells were incubated in 5% CO_2_ at 95% humidity and 37°C. When cell clones had been formed, the culture medium was replaced with fresh medium. The medium was changed every 3 days. When the hUSCs reached 70–80% confluence, they were digested using 0.25% trypsin/EDTA and passaged into new flasks at a ratio of 1:1 at primary passage and at a ratio of 1:3 at other passages. The hUSCs at P3, P5, and P7 were used in this study.

### Cell morphology, proliferation, colony-forming, migration, and multidifferentiation analyses

#### Cell morphology and proliferation

Cell images were captured with an IX71 microscope imaging system (Olympus, Japan). Cell proliferation was assessed using Cell Counting Kit-8 (CCK-8) tests (Dojindo, Japan) according to the manufacturer’s instructions. Briefly, 2 × 10^3^ hUSCs and hBMSCs at passages P1, P3, P5, and P7 were seeded onto a 96-well plate. After culturing for 1, 3, 5, 7, and 9 days, 10 μL CCK-8 was added into a 100-μL culture medium. The plate was incubated at 37°C for 2 h, and the optical density at 450 nm was measured immediately with a spectrophotometer (Biotek Instruments, USA).

#### Cell colony-forming

For colony-forming analysis, hUSCs and hBMSCs were seeded at a density of 1 × 10^3^ onto a 24-well plate. After 14 days of incubation, cells were fixed with 10% paraformaldehyde and stained with 0.5% crystal violet (Solarbio) for 30 min. Cells were washed with distilled water and captured. The stained colony was dissolved in 10% acetic acid, and the optical density (OD) was measured at 562 nm.

#### Cell migration

A scratch wound closure assay and transwell migration assay were performed to evaluate the migration ability of hUSCs and hBMSCs. For the scratch wound closure assay, cells (5 × 10^5^ cells/well) were seeded onto 6-well plates. A scratch of ~0.5 mm was created using a sterile pipette tip when cells reached 80–90% confluence at 12 h of culture. Each well was washed twice with PBS. Cell migration into the scratch was captured at 0, 6, 12, and 24 h, and results were analyzed with ImageJ software (National Institutes of Health, USA, version 1.47t). For the transwell migration assay, a 200-μL culture medium containing 2 × 10^4^ cells was placed in the upper chamber of a 24-well transwell plate (pore size: 8 mm, Corning, USA), and 600 μL culture medium was placed in the lower chamber. After 24 h of culture, the upper surface of the transwell membrane was scraped with a cotton swab to remove the adherent cells. The cells that had migrated to the lower side of the membrane were fixed with 4% paraformaldehyde for 30 min and stained with 0.1% crystal violet for 10 min. The migrated cells were counted in five random high-power (200×) microscopic fields in each well.

#### Cell Multidifferentiation

In order to analyze the tri-potentiality of hUSCs and hBMSCs, the abilities of osteogenic and adipogenic differentiation were verified by two-dimensional (2D) plate induction culture, and the chondrogenic differentiation ability was verified by a three-dimensional (3D) pellet formation experiment and 2D plate induction culture, as previously described. Briefly, for 2D induction culture, cells were seeded at a density of 3 × 10^4^ cells/well in 24-well plates in growth medium. At 80% confluence, the medium was replaced by each differentiation medium (Cyagen). The medium was changed every 3 days. Alkaline phosphatase (ALP; Yeasen, Shanghai, China) staining was performed after 14 days of osteogenic induction, and the level of ALP activity was quantified using the AKP/ALP kit (Jiancheng, Nanjing, China). Alizarin red S (ARS; Cyagen) staining was performed after 21 days of osteogenic induction, and to quantify the calcium content, the calcium-bound alizarin red was dissolved into the solution by incubating the stained samples in 10% cetylpyridinium chloride (TCI, Shanghai, China) for 1 h; the absorbance at 562 nm was examined via spectrophotometer and the data were expressed as OD values normalized by cell number. Oil red O (ORO; Cyagen) staining was performed at 14 d of adipogenic induction, and the stained Oil red O was eluted with isopropanol; the OD value of the solution was measured at 520 nm, and Alcian blue (Cyagen) staining was performed after 21 days of chondrogenic induction.

After 21 days of chondrogenic induction, Alcian blue (Cyagen) staining, western blot analysis, and quantitative reverse transcription polymerase chain reaction (qRT-PCR) were performed. Western blot analysis was performed as previously described [23]. Chondrogenesis-related primary antibodies included Sox9 (rabbit, 1:1,000, GeneTex), Aggrecan (Agg; rabbit, 1:500, Proteintech, Rosemont, IL, USA), collagen I (Col I; rabbit, 1:800, Proteintech), and Col II (rabbit, 1:200, Proteintech). GAPDH (rabbit, 1:1,000, GeneTex, Irvine, CA, USA) was used as an internal control and the secondary antibody was goat anti-rabbit antibody (1:2,500, ZSBIO, Beijing, China). To detect chondrogenesis-related gene expression, qRT-PCR was performed as previously described [24]. Chondrogenesis-related genes included *Agg*, *Sox9*, and *Col II*. GAPDH was used as an internal control. All primers were synthesized by the Sangon Biotech (Shanghai, China) (Table 2).

**Table 2:** Primers used for qRT-PCR

Gene	Primer/probe	Sequence(5′ to 3′)
Aggrecan	Forward: Reverse:	GTGAAAGGTGTTGTGTTCCAC TGGGGTACCTGACAGTCTGAT
Sox9	Forward: Reverse:	GCGGAGGAAGTCGGTGAAGAAT AAGATGGCGTTGGGCGAGAT
Col II	Forward: Reverse:	CACGCTCAAGTCCCTCAACA TCTATCCAGTAGTCACCGCTCT
GAPDH	Forward: Reverse:	CAAGAAGGTGGTGAAGCAGG CACTGTTGAAGTCGCAGGAG

For 3D induction culture, 1 × 10^6^ cells at P3 were centrifuged for 5 min at 500×*g* after which the pellet was cultured in chondrogenic medium (Cyagen). The medium was replaced every 3 days. After 21 days of chondrogenic induction, the pellets were fixed in 4% paraformaldehyde (PFA) in PBS for 48 h and 15% EDTA for 2 weeks. The samples were washed in PBS, dehydrated through an alcohol gradient and embedded in paraffin blocks. Next, 5-μm-thick histological sections were cut at the center of the embedded samples, followed by staining with hematoxylin and eosin (H&E), Alcian blue, safranin O, Agg, and Col II.

### Preparation and characterization of ACM scaffolds

#### Preparation of ACM scaffolds

The decellularized process of cartilage slices and the preparation of ACM scaffolds were summarized in our previous report [25]. Briefly, after the decellularized treatment, the ACM suspension was stirred for 4 h at room temperature and added to a 5-mm-diameter and 3-mm-high cylindrical mold, followed by lyophilization for 24 h. The preliminary scaffolds were then crosslinked with 50 mM 1-ethyl-3-(3-dimethylaminopropyl) carbodiimide hydrochloride (EDC) and 20 mM *N*-hydroxy succinimide (NHS) (pH 4.5–5.0) in ethanol for 6 h at room temperature and then rinsed with ultrapure water 10 times by changing the liquid every 30 min to remove excess reagent. After freeze-drying for 24 h, we obtained the final scaffolds labeled ACM+, and scaffolds without crosslinking were labeled ACM–. All the scaffolds were sterilized using ethylene oxide prior to use.

#### Ultrastructure analyses

The morphology of scaffolds and unacellular cartilage slices was observed using S-4800 scanning electron microscopy (SEM; Hitachi, Kyoto, Japan). The samples were sputtered with gold for 60 s using SC7620 gold sputter–coating equipment (Quorum Technologies, UK). For each sample, we randomly selected three visual fields, and the pore size of each scaffold sample was measured using ImageJ software.

The average porosity of the two scaffold groups was measured using liquid displacement. We used hexane because it can permeate through scaffolds without swelling or shrinking the matrix. Dried scaffolds were immersed in a known volume (V1) of hexane in a graduated cylinder for 5 min, the total volume of hexane and the sample was recorded as V2, and after removing the scaffolds, the residual hexane volume was recorded as V3. The scaffold porosity was measured as [(V1 − V3)/(V2 − V3)] × 100.

#### Histological and biochemical evaluations

The two groups of scaffolds were stained with H&E, Masson’s trichrome (Masson), safranin O, and toluidine blue in order to observe the structural change. The GAG, DNA, and collagen contents of unacellular cartilage slices and the ACM– group were quantified using dimethylmethyleneblue (Sigma-Aldrich), the QuantiFluor® double-standard DNA (dsDNA) system (Promega Corporation, Madison, WI, USA), and the hydroxyproline assay kit (Jiancheng, Najing, China), respectively, according to instructions of the corresponding agents and kits. Briefly, samples were freeze-dried with a constant weight and digested in papain solution in a water bath for 24 h at 60°C. The papain solution was cleared by centrifugation at 10,000×*g* for 30 min, and the solution obtained was assayed using the aforementioned reagents and kits.

#### Hydrophilic, swelling, and mechanical properties

Scaffold hydrophilicity was analyzed using contact angle detection (Theta Flex, Biolin Scientific, Sweden) according to instructions. For swelling properties, scaffolds were immersed in ultrapure water for 24 h at room temperature. Excess water was removed, and the scaffolds’ wet weight (*W*_w_) was measured. The samples were dried in an oven at 65°C under vacuum overnight, and the scaffolds’ dry weight (*W*_d_) was measured. Finally, the scaffolds’ swelling ratio was calculated as follows: swelling (%) = [(*W*_w_ − *W*_d_)/*W*_d_] × 100.

The elastic modulus of the two scaffold groups was evaluated using compressive testing. Briefly, samples were made into a 10-mm-diameter and 7-mm-high cylinder using a mold. The elastic modulus, defined by the slope of the initial linear section of the stress–strain curve, was measured using 500 N force at a loading velocity of 1 mm/min (Instron, Norwood, MA, USA).

### Comparison of chondrogenesis-related biological behaviors between hUSCs and hBMSCs cultured on scaffolds

#### Cell viability, morphology, and proliferation tests

The scaffolds were placed in 48-well plates and soaked in growth medium for 24 h. The medium was removed, and 1 mL of growth medium containing 5 × 10^3^ hUSCs or hBMSCs was seeded onto scaffolds. The plates were incubated in 5% CO_2_ at 95% humidity and 37°C. The culture medium was replaced every 2 days. After 3 days of culture, the scaffolds were stained using the live−dead staining kit (Yisheng, Shanghai, China) according to the manufacturer’s instructions. The stained scaffolds were observed under a Lake Success laser scanning confocal microscope (Olympus, Japan), and the live–dead cell ratio was calculated. Excitation light originated from calcein-AM and propidium iodide. At the same time point, cells were dehydrated using a gradient series of ethanol/water solutions (10%, 20%, 35%, 50%, 70%, 85%, and 100%) and dried using the CO_2_ critical-point drying method. The SEM was operated at 5 kV to image the samples. After 1, 4, and 7 days of culture, cell proliferation was quantified using the CyQuant assay kit (Thermo Fisher Scientific) based on DNA fluorescence, according to the manufacturer’s instructions.

#### In vitro chondrogenesis

To evaluate in vitro chondrogenic differentiation of cells on scaffolds, the scaffolds were placed in a 48-well plate, and 1.5 × 10^5^ hUSCs or hBMSCs were seeded onto each scaffold. The cells were cultured in growth medium for 3 days and then in chondrogenic induction medium. After 21 days of chondrogenic induction, biochemical evaluations were performed. The DNA, GAG, and total collagen contents of the samples were quantified. After 7, 14, and 21 days of chondrogenic induction, qRT-PCR was performed to determine the chondrogenesis-related gene expression of cells cultured on scaffolds, and the genes included *Aggrecan*, *Sox9*, and *Col II*.

### Comparison of chondrogenesis in vivo

#### Animal surgery procedure

Thirty-two 3-month-old male New Zealand white rabbits were randomly assigned to four groups: Blank, ACM, hUSCs, and hBMSCs. Briefly, a scaffold loaded with 5 × 10^5^ hUSCs or hBMSCs was cultured in chondrogenic induction medium for 7 days before surgery. The rabbits were anesthetized using sodium pentobarbital. Critical-size cartilage defect models, 5 mm in diameter and 3 mm in length, were created on the patellar groove in bilateral knee joints, and different scaffolds were implanted into the defects. Rabbits implanted with scaffolds loaded with hUSCs or hBMSCs were labeled hUSCs or hBMSCs, respectively; rabbits implanted with scaffolds without cells were labeled ACM; and rabbits without implantation after surgery were labeled Blank.

#### Magnetic resonance imaging (MRI) and micro-computed tomography (μCT) analyses

MRI (Achieva 3.0T, Phlips, Netherland) scanning was performed after the rabbits in each group underwent anesthesia at 6 and 12 weeks postoperatively to evaluate cartilage repair, as previously described. After 6 and 12 weeks postsurgery, the rabbits were sacrificed with excessive sodium pentobarbital and the knee joints were harvested and fixed in 10% formalin for μCT imaging and histological analysis. 3D structures of the regenerated tissue within the defects were evaluated with μCT (SkyScan 1176, SkyScan, Aartselaar, Belgium). 3D reconstruction and bone volume fraction (bone volume [BV]/ tissue volume [TV]) for new bone formation were analyzed using the system software. The knee joint from rabbits without surgery were labeled untreated and used as the control group.

#### Inflammation and toxicity assays

At 6 weeks after implantation, the rabbits were anesthetized, and synovial fluid was collected and centrifuged at 5000 rpm for 15 min at 4°C. The inflammatory factor interleukin-1 (IL-1) and tumor necrosis factor-a (TNF-α) in the synovial fluid were analyzed using the enzyme-linked immunosorbent assay (ELISA; Neobioscience, Guangdong, China), according to the manufacturer’s instructions. Then, the rabbits were sacrificed by an overdose of anesthesia, and the synovium in front of the knee joint, liver, lungs, and kidneys were harvested and stained with H&E to evaluate the inflammation and toxicity response of scaffolds implanted in vivo.

#### Gross observation and histological evaluation

At 6 and 12 weeks’ postimplantation, specimens were harvested and the gross morphology was observed and photographed. Histological evaluations were performed using H&E, Masson’s trichrome, Col I, and Col II staining, as previously described. Histological staining results were quantified by Pineda points [26].

### Statistical analysis

All experiments were performed in triplicate, unless otherwise indicated. Data were expressed as means ± standard deviations (SDs). Statistical analysis was performed using SPSS Statistics 16.0 for Windows (Chicago, IL, USA) using one-way analysis of variance, followed by the Tukey’s multiple-comparison test to evaluate between-group differences. *P* < 0.05 was considered statistically significant.

## Results

### Morphology and proliferation of hUSCs and hBMSCs

**Fig. 1:**
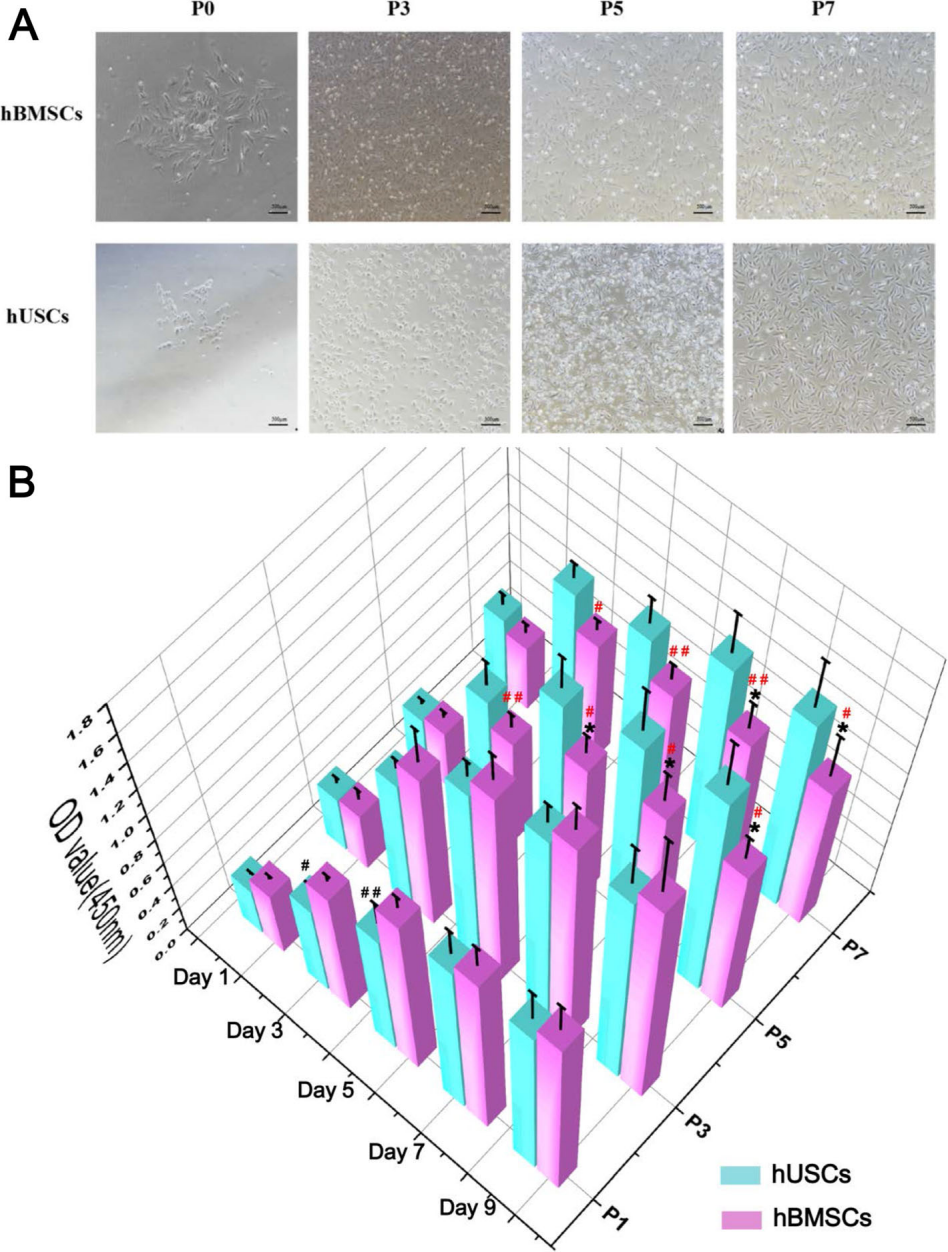
**a** Cell morphology of hBMSCs and hUSCs at P0, P3, P5, and P7. Scale bar = 500 μm. **b** Cell proliferation of hUSCs and hBMSCs at P1, P3, P5, and P7. *p < 0.05 and **p < 0.01, hBMSCs compared to hUSCs at the same passage; ^#^*p* < 0.05 and ^##^*p* < 0.01, hUSCs compared to hUSCs at P3; ^#^*p* < 0.05 and ^##^*p* < 0.01 in red, hBMSCs compared to hBMSCs at P3

After culturing for 5 to 10 days, adherent cells from urine and bone marrow began to form cell clones. At primary passage (P0), hUSCs had a spindle- or rice-like shape, and hBMSCs showed the similar spindle-shaped fibroblast-like morphology (Fig. 1a). With increased cell passages, the number of hUSCs with a rice-like shape gradually decreased, and the number of spindle-shaped cells gradually increased; at P7, most of hUSCs were spindle-shaped, and most of hBMSCs maintained the spindle shape, while a small number of cells displayed polygonal morphology at P5 and P7.

In comparing cell proliferation, hUSCs and hBMSCs (donor 1) both reached peak growth speed on day 3, and hUSCs had higher proliferative capacities in contrast to BMSCs at P5 and P7 after day 5. For hUSCs, no significant difference was found among different passages except for P1; while for hBMSCs, cells at P3 had the highest growth rate from day 3 to day 9, and cells at P5 and P7 had a significantly lower growth rate compared to P3 (Fig. 1b).

### Cell colony-forming and migration

**Fig. 2:**
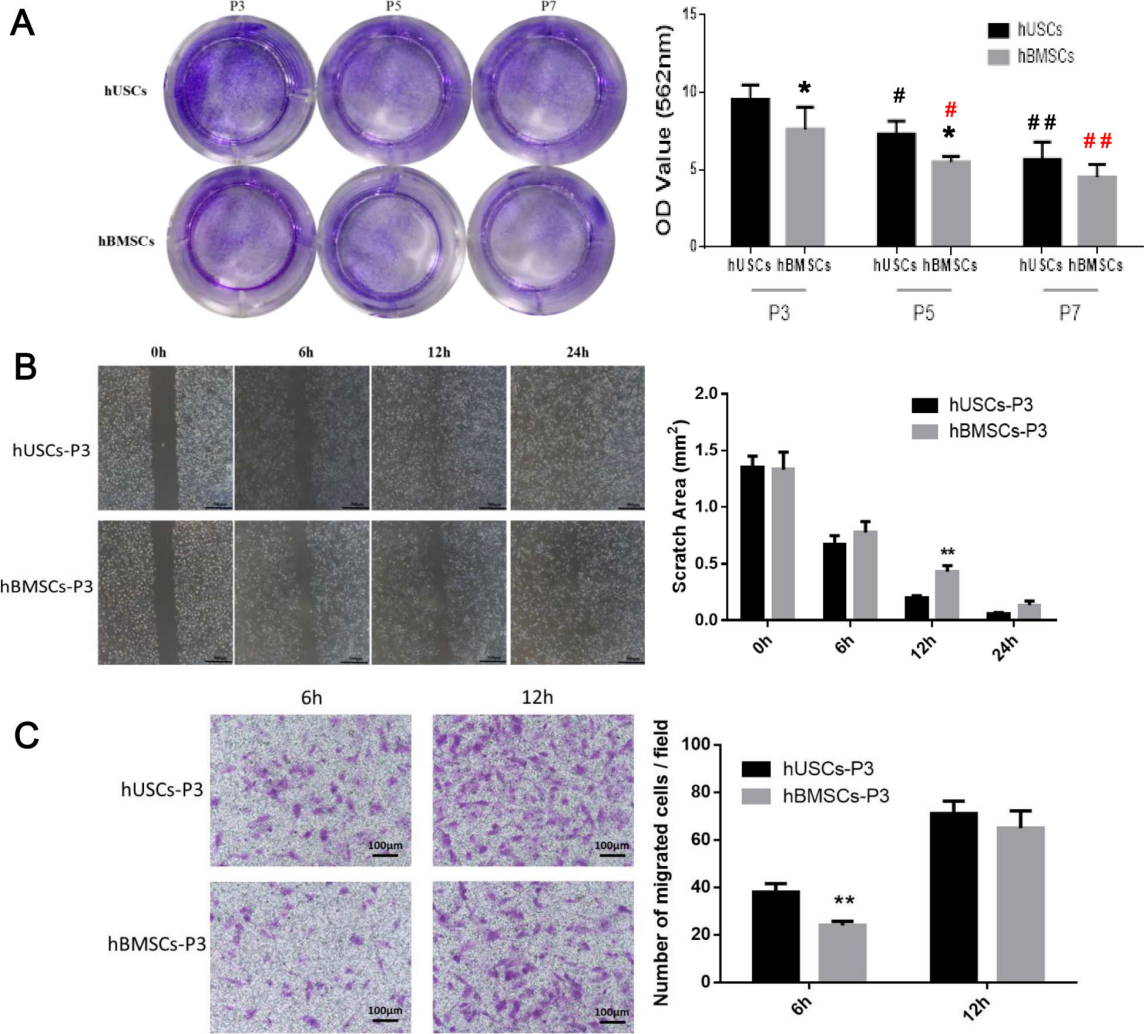
**a** Colony formation analysis of hUSCs and hBMSCs at P3, P5, and P7. **b** Scratch wound closure assay of hUSCs and hBMSCs at P3. Scale bar = 500 μm. **c** Transwell migration assay of hUSCs and hBMSCs at P3. Scale bar = 100 μm. *p < 0.05 and **p < 0.01, hBMSCs compared to hUSCs at the same passage; ^#^*p* < 0.05 and ^##^*p* < 0.01, hUSCs compared to hUSCs at P3; ^#^*p* < 0.05 and ^##^*p* < 0.01 in red, hBMSCs compared to hBMSCs at P3

In colony formation analysis, cells were obtained from donor 1. hUSCs showed more clone forming units compared to hBMSCs at P3 and P5 (Fig. 2a). hUSCs and hBMSCs at P3 both presented the highest colony-forming capacity compared to cells at P5 and P7. To evaluate cell migration, hUSCs and hBMSCs at P3, which showed better proliferative and colony-forming capacities, were selected for follow-up experiments. A scratch wound closure assay was performed to detect the migration capacity of cells on the horizontal plane, and hUSCs showed a smaller scratch area at 12 h compared to hBMSCs (Fig. 2b). In the transwell migration assay, more hUSCs had migrated vertically to the lower side of the membrane through the micropores at 6 h. Therefore, hUSCs at P3 showed higher migration capacity compared to hBMSCs (Fig. 2c).

### Multilineage differentiation potential

For osteogenic and adipogenic capacity analyses, cells were obtained from donor 2. The ALP activity and calcium content of hBMSCs at P3 were higher than hUSCs at P3 (Figure S1A, B). hUSCs and hBMSCs at P3 both displayed the higher ALP activity and calcium content compared to cells at P7. These quantitative results indicated that hBMSCs had higher osteogenic capacity compared to hUSCs at P3, and hUSCs and hBMSCs at P3 showed stronger osteogenic potential compared to cells at P5 and P7. Similarly, hBMSCs at P3 showed higher lipid droplet content compared to hUSCs at P3 and P5, indicating hBMSCs have better adipogenic capacity compared to hUSCs at the same passage (Figure S1C). Both hUSCs and hBMSCs at P3 had stronger adipogenic capacity compared to cells at P5 and P7.

**Fig. 3:**
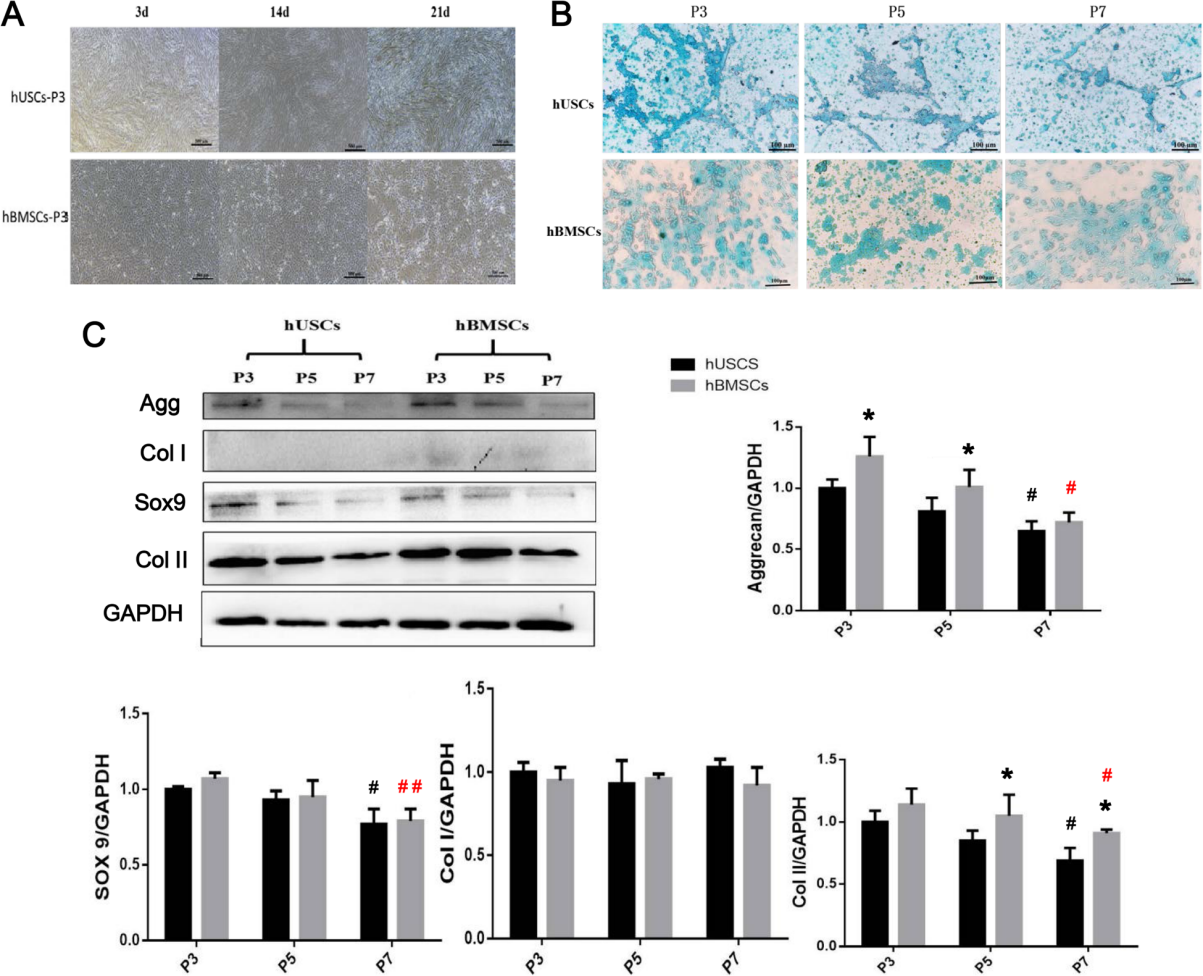
**a** Cell morphology of hUSCs and hBMSCs at P3 after 21 days of 2D chondrogenic differentiation. Scale bar = 500 μm. **b** Alcian blue staining results of hUSCs and hBMSCs at P3, P5, and P7 after 21 days of 2D chondrogenic differentiation. Scale bar = 100 μm. **c** The expression of Agg, Col I, Sox9, and Col II analyzed using western blot after 21 days of 2D chondrogenic differentiation. *p < 0.05 and **p < 0.01, hBMSCs compared to hUSCs at the same passage; ^#^*p* < 0.05 and ^##^*p* < 0.01, hUSCs compared to hUSCs at P3; ^#^*p* < 0.05 and ^##^*p* < 0.01 in red, hBMSCs compared to hBMSCs at P3

**Fig. 4:**
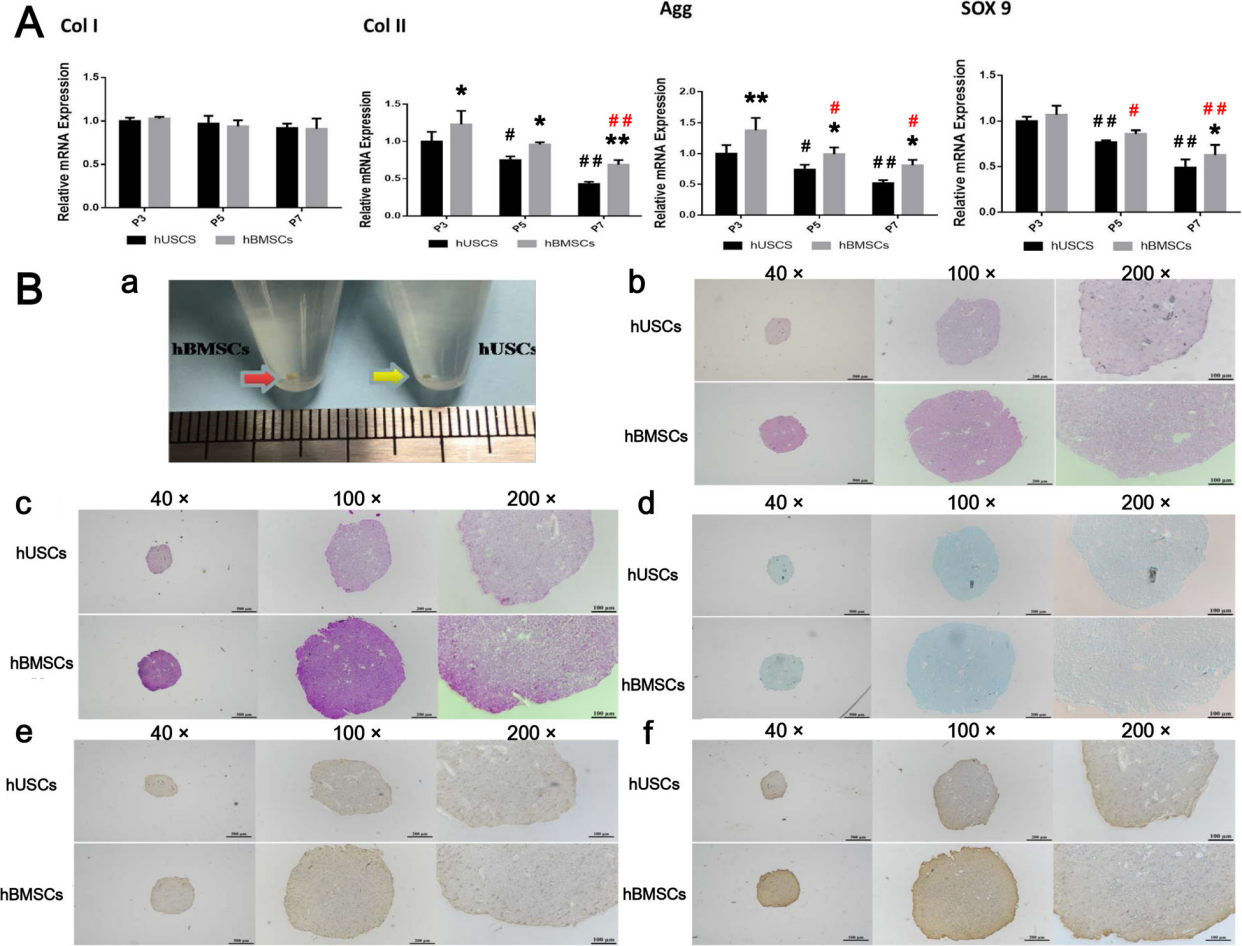
**a** Detection of the mRNA levels of selected chondrogenic markers in hUSCs and hBMSCs at P3, P5, and P7 after 21 days of 2D chondrogenic differentiation. **b** The gross observation (**a**) and histological staining results (**b**–**f**) of the hUSCs and hBMSCs pellets after 21 days of 3D chondrogenic differentiation. **b** H&E, **c** safranin O, **d** Alcian blue, **e** Agg, and **f** Col II. The scale bars are 500 μm, 200 μm, and 100 μm in images with different magnifications (40 ×, 100 ×, and 200 ×), respectively. *p < 0.05 and **p < 0.01, hBMSCs compared to hUSCs at the same passage; ^#^*p* < 0.05 and ^##^*p* < 0.01, hUSCs compared to hUSCs at P3; ^#^*p* < 0.05 and ^##^*p* < 0.01 in red, hBMSCs compared to hBMSCs at P3

After 21 days of 2D chondrogenic differentiation, hUSCs and hBMSCs (donor 3) had similar morphological changes at all passages. Cells at P3 were taken as an example and are shown in Fig. 3a. On day 3, the cells tended to grow and gather together, and the morphology of the cells gradually flattened and lengthened; on day 14, the aggregative cells formed spiral clusters; on day 21, the clustered cells did not further gather but formed white tissue-like cell masses. The Alcian blue staining results of these masses were strongly positive (Fig. 3b). At the same time, chondrogenesis-related proteins were observed using western blot (Fig. 3c). The Agg expression of hBMSCs at P3 and P5 was higher than that of hUSCs at the same passage. The Col II expression of hBMSCs at P5 and P7 was higher than that of hUSCs at the same passage. Both of hUSCs and hBMSCs at P3 displayed notably higher expressions of Agg, Sox9, and Col II compared to cells at P7. There was no significant difference in Col I expression between hUSCs and hBMSCs at all passages. Similar results were also obtained from the qRT-PCR results (Fig. 4a). These results indicated that hUSCs and hBMSCs at P3 had better chondrogenic ability compared to cells at other passages.

After 21 days of 3D chondrogenic differentiation, hUSCs and hBMSCs (donor 3) at P3 aggregated and formed a pellet, and the pellet diameter of hUSCs (5.42 ± 0.31 mm) was smaller than that of hBMSCs (7.43 ± 0.74 mm) (*p* < 0.05) (Fig. 4b). The staining results of safranin O, Alcian blue, Agg, and Col II in hUSCs and hBMSCs pellets were all positive, and hBMSCs formed larger cell pellets containing more chondrocyte extracellular matrix compared to hUSCs. Examination of chondrogenesis-related ability via 2D or 3D chondrogenic differentiation indicated that hBMSCs has stronger chondrogenic capacity compared to hUSCs at the same passage.

### Scaffold characterization

Biochemical analyses were performed to evaluate acellular results (Figure S2A). After decellularization, the scaffolds preserved most of the extracellular matrix (ECM) peculiar to hyaline cartilage, such as GAGs and collagen. In addition, there were no chondrocytes, cell fragments, or DNA components, indicating successful fabrication of the ACM.

SEM results showed that the internal structure of scaffolds with cross-linking (ACM+) had a specific direction and more interconnected pores compared to scaffolds without cross-linking (ACM–) (Figure S2B). There was no significant difference in pore size, porosity, and swelling rate between ACM– and ACM+, which indicated that the cross-linking process did not change these properties of ACM scaffolds (Figure S2C). Histological staining results showed that the internal structure of ACM– had an irregular distribution, while that of ACM+ had a more regular distribution (Figure S3A). ACM+ preserved most of the ECM after cross-linking, including collagen and GAGs. ACM+ scaffolds were also rich in interconnected pores compared to ACM– scaffolds.

Both ACM– and ACM+ were superhydrophilic (dripping water could be quickly absorbed by all scaffolds), and the contact angle was 0° (Figure S3B). The compressive elastic modulus of ACM+ was slightly higher than that of ACM–, but there was no significant difference between the two types of scaffolds (Figure S3C). These characterization results indicated that ACM+ scaffolds had a more orderly and interconnected structure and a slightly higher elastic modulus, which could favor nutrient exchange and promote cell growth and differentiation in the local environment.

### Chondrogenesis-related biological behaviors in vitro

**Fig. 5:**
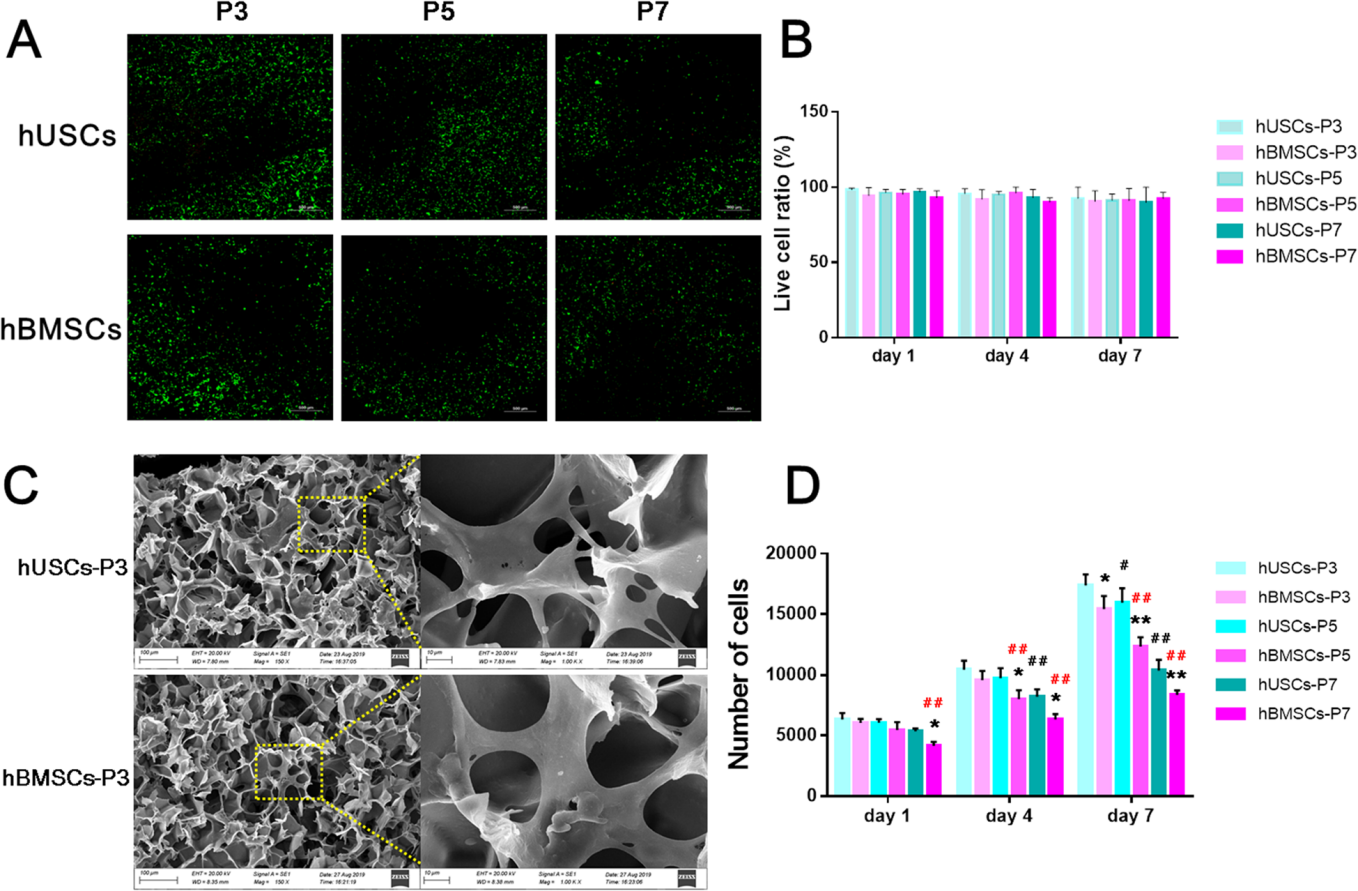
**a** Live (green) and dead (red) cells of hUSCs and hBMSCs cultured on scaffolds after 3 days, via live-dead staining. **b** Live cell ratio in live-dead staining results. **c** SEM images of cell adhesion on scaffolds after seeding for 3 days. The scale bars are 100 μm in low magnification images and 10 μm in high magnification images. **d** Cell proliferation of cells cultured on scaffolds after 1, 4, and 7 days, via the detection of DNA content. *p < 0.05 and **p < 0.01, hBMSCs compared to hUSCs at the same passage; ^#^*p* < 0.05 and ^##^*p* < 0.01, hUSCs compared to hUSCs at P3; ^#^*p* < 0.05 and ^##^*p* < 0.01 in red, hBMSCs compared to hBMSCs at P3

For the analyses of live–dead staining, cell proliferation, and SEM, cells were obtained from donor 4. Live–dead staining was performed to evaluate cell viability on scaffolds (Fig. 5a, b). After cells were cultured on scaffolds for 3 days, the number of living cells decreased with the increase of cell passage, but the live-cell ratios did not change significantly, which were all above 90%. For cell proliferation analysis, hUSCs and hBMSCs were implanted into the ACM+ scaffolds (Fig. 5d). On day 1, we found no significant difference in cell numbers between hUSCs and hBMSCs at P3 and P5, while the number of hBMSCs at P7 was lower than that of hUSCs. On days 4 and 7, hUSCs showed a significantly higher proliferative rate compared to hBMSCs at P5 and P7, and the number of hUSCs at P3 was the highest among the six groups of cells. Both of the numbers of hUSCs and hBMSCs at P3 were higher than cells at P5 and P7. The SEM images showed that the ACM+ scaffolds supported the attachment and growth of hUSCs and hBMSCs at P3, because both types of cells had well-spread morphology with lamellipodia/filopodia extending into the scaffolds. These results indicated that the ACM+ scaffolds had good biocompatibility and promoted cell attachment and growth. In addition, hUSCs and hBMSCs at P3 had the highest proliferative rate, and hUSCs had better proliferative capacity compared to hBMSCs at the same passage.

**Fig. 6:**
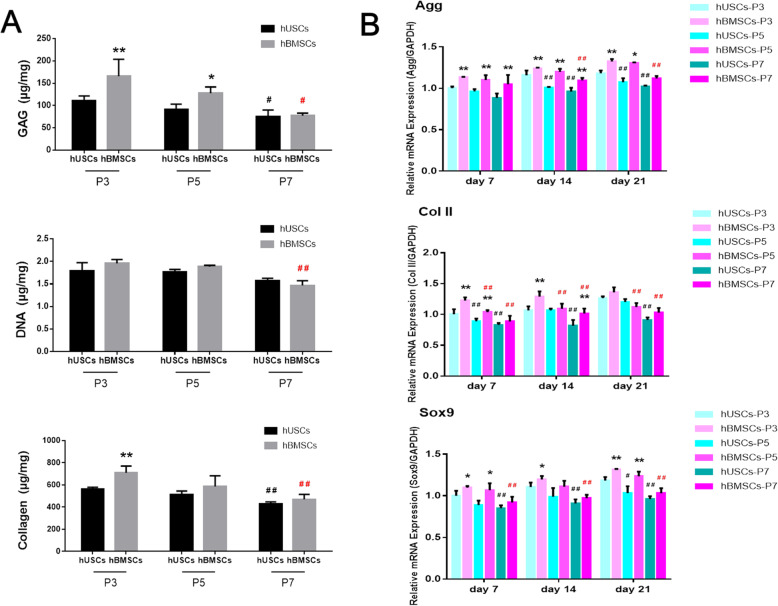
**a** Biochemical assay of samples after 21 days of chondrogenic induction, including the contents of GAG, DNA, and total collagen. **c** Detection of the mRNA levels of selected chondrogenic markers (*Agg, Col II, and Sox9*) in hUSCs and hBMSCs cultured on scaffolds after 7, 14, and 21 days of chondrogenic induction. *p < 0.05 and **p < 0.01, hBMSCs compared to hUSCs at the same passage; ^#^*p* < 0.05 and ^##^*p* < 0.01, hUSCs compared to hUSCs at P3; ^#^*p* < 0.05 and ^##^*p* < 0.01 in red, hBMSCs compared to hBMSCs at P3

For chondrogenic capacity analyses, cells were obtained from donor 5. After 21 days of chondrogenic induction, the GAG and total collagen contents in hUSCs at P3 were significantly lower compared to the hBMSCs at P3 (Fig. 6a). There was no significant difference in DNA contents between hUSCs and hBMSCs at the same passage. The GAG and total collagen contents in hUSCs and hBMSCs at P3 were higher compared to cells at P7. In comparison of chondrogenic genes, hBMSCs at P5 and P7 had notably higher gene expression in *Agg*, *Col II*, and *Sox9* compared to hUSCs at the same passage (Fig. 6b). Cells at P3 displayed higher mRNA levels in these genes compared to cells at P5 and P7. These chondrogenic examinations in vitro showed that hBMSCs cultured on scaffolds had better chondrogenic capacity compared to hUSCs at the same passage, and both of the two types of cells had the strongest chondrogenic potential at P3.

### Chondrogenesis-related biological behaviors in vivo

Cells used in vivo were obtained from donor 6. Inflammation in the surrounding tissues and toxicity response of internal organs after implantation were evaluated to check the biosafety of scaffolds loaded with hUSCs and hBMSCs. At 6 weeks after surgery, there was no significant neutrophil infiltration in all of the four groups according to the H&E staining images of the synovium in the joint (Figure S4A). There was also no significant difference in the contents of IL-1 and TNF-α in synovial fluid among the four groups (Figure S4B). In addition, the internal organs were evenly stained, and no obvious toxic reactions such as blood cell oozing, inflammation, and cell necrosis were observed (Figure S4C). These results confirmed that ACM+ scaffolds loaded with hUSCs and hBMSCs had good biocompatibility and did not induce significant immune reactions.

**Fig. 7:**
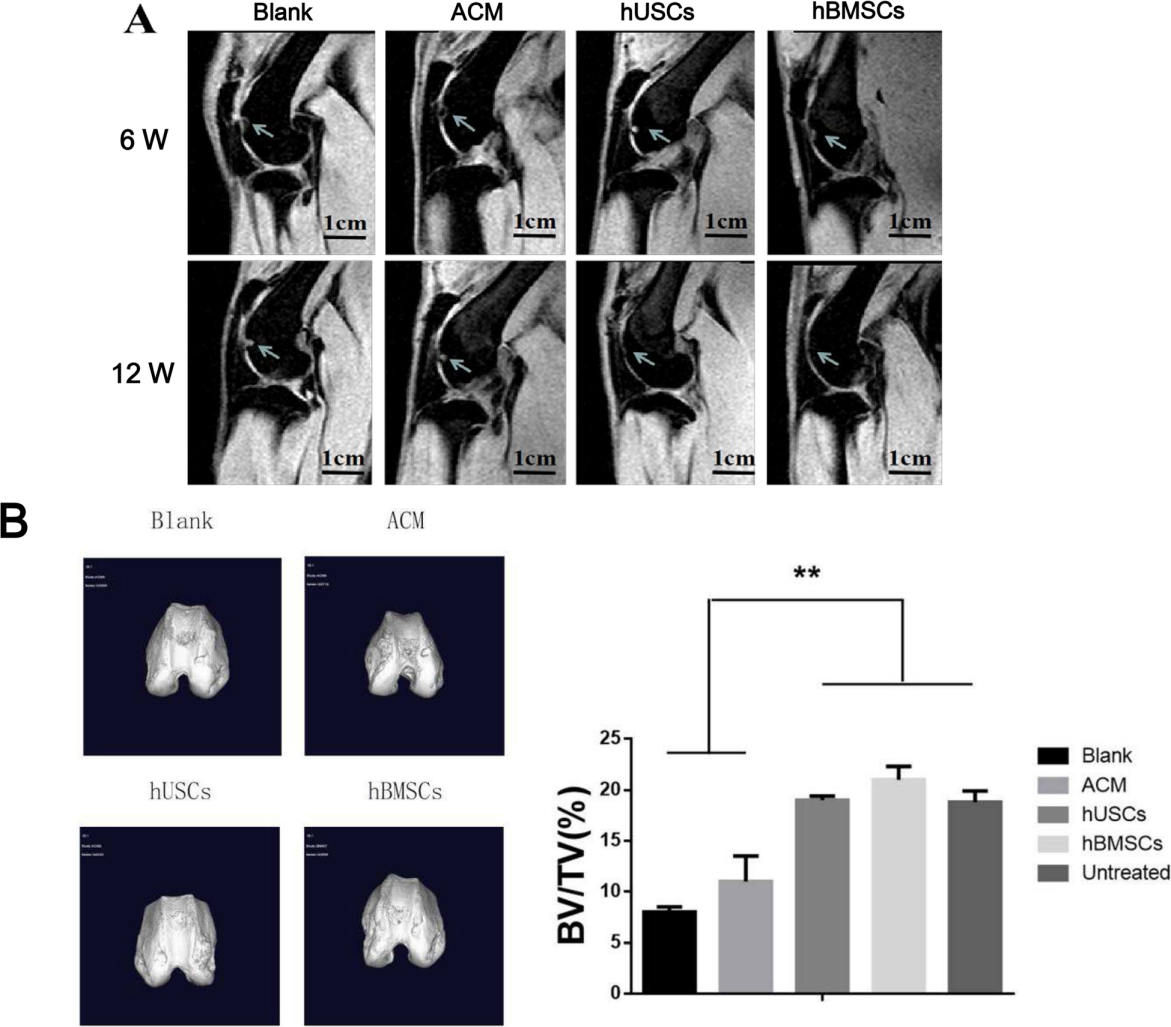
**a** T2 images of the knees at 6 and 12 weeks post-surgery (fat suppression; arrow, repaired sites of articular cartilage; scale bar = 1 cm). **b** μCT results of the specimens and the bone volume faction at repaired sites at 12 weeks post-surgery. Statistically significant differences are indicated with *p < 0.05 and **p < 0.01

As shown in Fig. 7a, at 6 weeks postoperatively, the defects in the groups with scaffold implantation were partially filled with tissue of low signal intensity, while the defect in the Blank group was poorly filled with tissue of high signal intensity. At 12 weeks, the injuries in hUSC and hBMSC groups were fully filled with tissue. The repaired tissue had a smooth surface, and the signal intensity of the tissue was similar to that of the normal cartilage. The high-signal intensity at the repaired site in the ACM group was lower than before, but there was still some defect area. The Blank group had the worst repair effect.

The μCT results indicated that the bone contents at the repaired site in Blank and ACM groups were notably lower compared to hUSCs, hBMSCs, and untreated groups at 12 weeks postoperatively (Fig. 7b). The implantation with scaffolds loaded with cells promoted bone formation at the bottom of the defect area. There was no significant difference in the bone volume faction at repaired sites among hUSCs, hBMSCs, and untreated groups, indicating that ACM+ scaffolds loaded with hUSCs or hBMSCs could prevent subchondral bone loss at the cartilage injury sites.

**Fig. 8:**
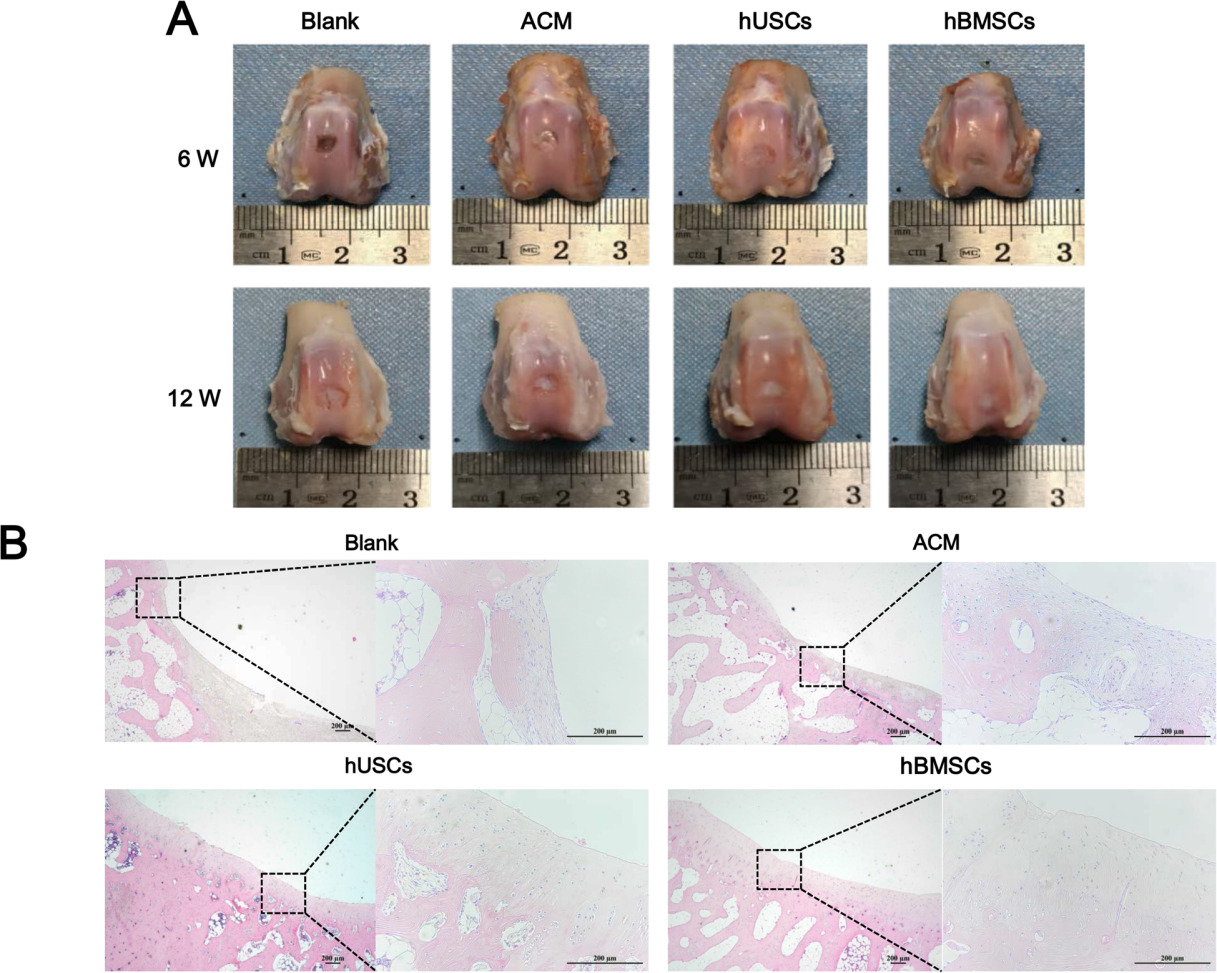
**a** Knee joint specimens from four groups at 6 weeks and 12 weeks of implantation. **b** Images of H&E staining of specimens at 12 weeks of implantation; scale bar = 200 μm

Specimens were collected 6 and 12 weeks postimplantation (Fig. 8a). At week 6 postsurgery, there was no obvious tissue growth into the defect in the Blank group, and the defects in groups with scaffold implantation were partially filled with tissue accompanied by scaffold degradation. At 12 weeks postoperatively, the defect in the Blank group was covered by some fibrous scar tissue, and the height of the repaired tissue was obviously lower than the surrounding cartilage. In the ACM group, the newly formed tissue had a rough surface and was poorly integrated with the surrounding tissue. Most of the defects in the hUSCs and hBMSCs groups were filled with transparent tissue that integrated well with the surrounding cartilage and had a smooth surface. All of the implanted scaffolds were completely degraded.

Histological images of H&E staining at 6 weeks postimplantation showed that the scaffolds in each group were almost degraded (Figure S5). The Blank and ACM groups had fewer new tissues, which were poorly integrated with surrounding tissue. There was more new tissue in the hUSCs and hBMSCs groups, but the new chondrocytes were disorderly arranged. The H&E staining results at 12 weeks postimplantation indicated numerous new chondrocytes accompanied by a thick, mature cartilage matrix in the hUSCs and hBMSCs groups (Fig. 8b). The four-layer structure (superficial, mid, dip, and calcified zones) of the hyaline cartilage in the new tissue was clear. There was no obvious boundary between the new and host tissues, and the repaired defect area was smooth. In the ACM group, we observed some new cartilage tissue, which was thin with an incomplete four-layer structure and poorly integrated with the surrounding tissue. In the Blank group, there was little new tissue and positive staining of the ECM, and the defect was mainly repaired with fibrous tissue and fibrocartilage. These results indicated that hUSC and hBMSC groups had a better cartilage repair effect.

**Fig. 9:**
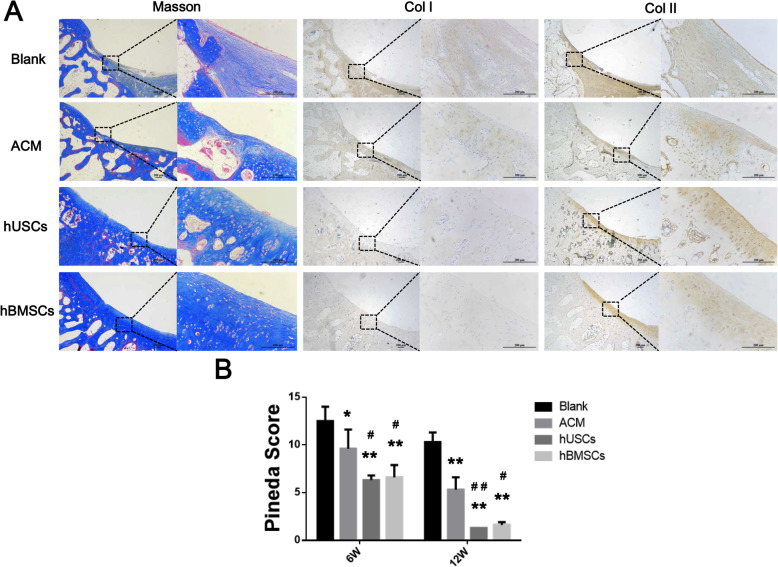
**a** Images of Masson, Col I, and Col II staining of specimens at 12 weeks postimplantation; scale bar = 200 μm. **b** Pineda points of specimens from four groups at 12 weeks postimplantation. *p < 0.05 and **p < 0.01, compared to the Blank group; ^#^*p* < 0.05 and ^##^*p* < 0.01, compared to the ACM group

Masson, Col I, and Col II staining were also performed at 6 (Figures S5, S6) and 12 weeks postimplantation (Fig. 9a). At 6 weeks after implantation, the new tissue in the Blank group had the least collagen content. For the ACM group, there was a large amount of Col II formation at the bottom of the defect area but less on the surface of the new tissue. The hUSC and hBMSC groups had more new tissue containing a lot of Col II. At 12 weeks postimplantation, a large amount of new collagen tissue bridged the defect area in the hUSC and hBMSC groups, followed by the ACM group, and Col II was dominant in these three groups. Repaired tissue in the Blank group had the lowest Col II content and had a relatively higher Col I content, which suggested that the newly formed tissue was composed of fibrocartilage or fibrous tissue. Col II is the main component of the hyaline cartilage ECM, so this result further proved that cartilage defects in the ACM, hUSC, and hBMSC groups are mainly repaired by the hyaline cartilage. As shown in Fig. 9b, the Blank group had the highest Pineda score, followed by the ACM group. Both the hUSC and hBMSC groups had lower Pineda scores, and there was no significant difference between them. These results indicated that hUSCs or hBMSCs cultured on ACM+ scaffolds promoted cartilage regeneration, and the two types of cells had similar cartilage repair effects in vivo.

## Discussion

Cartilage tissue engineering is considered to be one of the ideal methods to develop treatment for cartilage injuries [18]. Stem cells are often used as seed cells [27]. However, the process of clinical transformation from tissue engineering cartilage is often limited by the shortcomings of traditional stem cells, such as invasive isolation, few sources, and so on [9, 10]. On the other hand, hUSCs have the advantages of noninvasive isolation, fast and stable proliferation, and ability for repetitive isolation from the urine of the same individual [28, 29]. In this study, we verified the potential of hUSCs to replace traditional stem cells for cartilage repair using chondrogenesis-related experiments in vitro and in vivo.

Morphologically, hUSCs presented two distinct subpopulations including spindle shape and rice-like shape, and the proportion of spindle-shaped hUSCs increased with the increase of cell passages while the proportion of rice-shaped hUSCs decreased. As previously reported, the two subpopulations showed similar clone forming efficiency, but spindle-shaped hUSCs had greater capacities for proliferation, migration, and osteogenic and adipogenic differentiation while rice-shaped hUSCs had greater potential for chondrogenic differentiation [30]. In this study, considering the comprehensive biological performance of stem cells in promoting cartilage regeneration and multiple passages to obtain different subpopulations of hUSCs, we did not divide hUSCs into subgroups for research but compared the chondrogenesis-related biological behaviors of hUSCs and hBMSCs obtained from the same individual from P0. The hBMSCs showed a spindle-shaped fibroblast-like morphology, which is considered to be a typical character of mesoderm-origin mesenchymal stem cells [31]. At P7, most of hUSCs were spindle-shaped, and a small number of hBMSCs displayed polygonal morphology that might lose the typical biological characteristics of stem cells [32]. Therefore, we chose P7 as a maximum passage in our study. The proliferation experiment showed that the proliferative rate of hBMSCs at P7 was notably lower than that at P3, and the hUSCs and hBMSCs at P3 had stronger colony-forming capacity compared to cells at P7. The proliferative capacity of hUSCs at P5 and P7 was higher compared to hBMSCs, while there was no significant difference between hUSCs and hBMSCs at P3, indicating a more stable and efficient proliferation in hUSCs. These results indicated that the culture of hUSCs has lower cell-density dependence, and we found that only a few colonies (one to two) are enough for sustainable proliferation in primary culture.

In comparing multilineage differentiation, hBMSCs at P3 presented higher osteogenic and adipogenic capacities compared to hUSCs. At P7, there was no significant difference, because hUSCs have different subpopulations with different shapes, which may have different capacities of differentiation, and the proportion of spindle-shaped hUSCs increased with the increase in cell passage [33]. The expressions of chondrogenesis-related proteins and genes were higher in hBMSCs compared to hUSCs at the same passage, and more chondrocyte ECM was found in hBMSCs pellets compared to hUSCs pellets. These results indicated that hBMSCs had better capacities for multilineage differentiation compared to hUSCs at the same passage, and hUSCs and hBMSCs at P3 presented stronger differentiation potential compared to cells at other passages.

As previously reported, hUSCs showed lower osteogenic and chondrogenic differentiation ability compared to hBMSCs or hADSCs in vitro, and there is no comparative study on the repair of cartilage defects in vivo between hUSCs and other traditional stem cells [14, 15]. In this study, we fabricated ACM+ scaffolds loaded with hUSCs or hBMSCs for comparing cartilage repair effects in vivo. The ACM from porcine articular cartilage retains many bioactive factors and most of the ECM components, which promote the chondrogenic differentiation of stem cells and maintain the chondrocyte phenotype [34, 35]. In addition, because of the removal of cells and DNA components, the ACM can be used for allogeneic and heterogeneous cartilage repair, greatly enriching the source of scaffold materials. The results of scaffold characterization indicated that our improved decellularized method preserved ECM components from cartilage slices as much as possible while removing the cells and DNA components. The scaffolds after cross-linking had a more interconnected and orderly internal structure and were superhydrophilic, which could favor nutrient exchange and promote cell growth and differentiation in the local environment [36]. The physical properties of ACM+ scaffolds did not change a lot after cross-linking, and ACM+ scaffolds had a slightly higher elastic modulus, which might be more suitable for the mechanical requirement of tissue-engineered cartilage compared to ACM– scaffolds [37].

After cells were cultured on ACM+ scaffolds, chondrogenesis-related experiments were also performed. There was no significant difference in the live-cell ratio, indicating good biocompatibility of ACM+ scaffolds, and the scaffolds could promote cell adhesion and proliferation with the appropriate pore size and porosity. Examinations of biochemical assays and qRT-PCR showed similar results to the aforementioned experiments, which means hBMSCs cultured on scaffolds had higher chondrogenic capacities compared to hUSCs at the same passage, and cells at P3 had greater chondrogenic potential compared to cells at other passages. However, cartilage repair in vivo is a complex process, and its effectiveness is determined by many factors, such as the immune response, the local nutrition supply, and the interaction between host cells and implants [38, 39]. As previously reported, hUSCs differ from some traditional stem cells in immune regulation, and the expressions of CD80 and CD86 in hUSCs were lower than in hBMSCs [28, 39]. In addition, hUSCs can reduce T cell activation and increase the expression of IL-6 and IL-8, thus reducing the risk of immune rejection and accelerating tissue repair [28]. Moreover, many studies have reported that the role of stem cells in promoting tissue repair after implantation mainly depends on their paracrine secretions to regulate the biological behaviors of host cells [40, 41]. Therefore, experiments in vivo were carried out in our study to comprehensively compare the effects on cartilage regeneration between hUSCs and hBMSCs.

The detection of inflammatory factors and the histological evaluations of joint synovia and internal organs showed that the scaffold had good biocompatibility, and the two types of transplanted human-derived cells did not cause obvious immune rejection, indicating the low immunogenicity of the hUSCs and hBMSCs. As the MRI and histological results showed, the area of newly formed cartilage tissue at repaired sites occurred in the order of hUSCs = hBMSCs > ACM > Blank. Histological results indicated that the newly formed cartilage in groups with scaffolds was mainly composed of hyaline chondrocytes, and the application of hUSCs and hBMSCs can further improve the repair effect in rabbit articular cartilage. At 6 weeks postimplantation, the newly formed Col II in the ACM group was mostly aggregated at the bottom of the defect area, while Col II in the new tissue of the hUSC and hBMSC groups was greater and evenly distributed. This may be because human-derived stem cells promoted cartilage regeneration and accelerated the degradation and remodeling of the scaffold material. These cells could convert the ECM components in the scaffold into new cartilage tissue more efficiently and adjust the distribution of tissue components. At 12 weeks postimplantation, the cartilage defect area was almost bridged by the repaired hyaline cartilage in hUSC and hBMSC groups. Differing from the results in vitro, there was no significant difference in the effect of cartilage repair between hUSCs and hBMSCs. This result suggested that when stem cells are implanted into the body for tissue repair, they may mainly act as the driving force for the host to initiate and accelerate repair rather than differentiate into the corresponding tissue cells. This process may be achieved with paracrine secretions that could regulate the local immune response, mobilize the corresponding repair cells, and improve the ability of cell proliferation and differentiation into the corresponding tissue. Furthermore, in the results of μCT, we found that the bone mass at cartilage defect sites was well preserved due to the application of hUSCs or hBMSCs, which also shed light on the treatment of the combined injuries of bone and cartilage in some diseases such as arthritis with severe subchondral bone wear.

Nevertheless, the current study has several limitations. First, because of the different media which can maintain the stable and rapid proliferation of hUSCs and hBMSCs, respectively, the different cellular microenvironment provided by the media may affect cellular behaviors in vitro. In future studies, it may be considered to optimize the components of the media or supplement the media with additional growth factors to provide a same cellular microenvironment. Second, we focused on the differences between cellular behaviors of two types of cells from the same donor. However, there are indeed variations in cellular behaviors of the same stem cells from different donors. The interindividual variations also need to be explored in future studies. Third, because the main purpose of this study was to compare the difference of chondrogenesis-related biological behaviors between hUSCs and hBMSCs, the scaffold material used in this study can still be further improved. Finally, the tumorigenesis of hUSCs or hBMSCs is a major issue which should be investigated further in future studies before clinical applications. Overall, hUSCs can be considered to be an alternative to traditional stem cells for cartilage repair, but the difference of the capacity of hUSCs and hBMSCs to promote cartilage regeneration in vivo and in vitro needs to be further explored using cell tracking, genome analysis, and other experiments.

## Conclusion

We successfully isolated hUSCs and hBMSCs from the same individual. In in vitro experiments, hUSCs had better capacities for proliferation, colony-forming, and migration compared to hBMSCs at the same passage, while hBMSCs had greater osteogenic, adipogenic, and chondrogenic abilities compared to hUSCs at the same passage. Both of hUSCs and hBMSCs at P3 had the strongest potential for proliferation, colony-forming, and multilineage differentiation compared to cells at other passages. We developed an ACM scaffold with good biocompatibility and internal structure after cross-linking that supported hUSC and hBMSC adhesion, proliferation, and chondrogenic differentiation. The ACM+ scaffolds loaded with hUSCs or hBMSCs both significantly promoted the repair of cartilage defects in the rabbit knee model at 12 weeks postimplantation. We expect that hUSCs can be an alternative autologous stem cell source for cartilage regeneration and other tissue reconstruction instead of traditional stem cells.

## Supplementary Information

**Additional file 1: Figure S1.** Osteogenic and adipogenic induction results of hUSCs and hBMSCs at P3, P5, and P7. (A) ALP staining and normalized ALP activity detected at 14 days of osteogenic induction. Scale bar = 200 μm. (B) ARS staining and normalized Alizarin red intensity at 21 days of osteogenic induction. Scale bar = 200 μm. (C) ORO staining and normalized ORO intensity at 14 days of adipogenic induction. Scale bar = 100 μm. *p < 0.05 and **p < 0.01, hBMSCs compared to hUSCs at the same passage; ^#^*p* < 0.05 and ^##^*p* < 0.01, hUSCs compared to hUSCs at P3; ^#^*p* < 0.05 and ^##^*p* < 0.01 in red, hBMSCs compared to hBMSCs at P3. **Figure S2.** (A) Biochemical assay of the unacellular cartilage slices and ACM– scaffolds, including the contents of DNA, GAG, and total collagen. (B) SEM images of unacellular cartilage slices, ACM–, and ACM+. The scale bar is 100 μm (100 ×) or 20 μm (600 ×). (C) The pore size, porosity, and swelling rate of ACM– and ACM+. Statistically significant differences are indicated with *p < 0.05 and **p < 0.01. **Figure S3.** (A) Histological staining results of ACM– and ACM+ scaffolds, including H&E, Masson’s, safranin O, and toluidine blue. The scale bar is 500 μm (40 ×) or 200 μm (100 ×). (B) Contact angle detection of ACM– and ACM+. (C) Compressive elastic modulus of ACM– and ACM+. Statistically significant differences are indicated with *p < 0.05 and **p < 0.01. **Figure S4.** (A) H&E staining images of the synovium in the joint at 6 weeks; scale bar = 50 μm. (B) IL-1 and TNF-α contents in synovial fluid at 6 weeks. Statistically significant differences are indicated with *p < 0.05 and **p < 0.01. (C) H&E staining images of livers, lungs, and kidneys at 6 weeks; scale bar = 500 μm. **Figure S5.** Images of H&E and Masson staining of specimens at 6 weeks of implantation. The scale bars are 500 μm in low magnification images and 200 μm in high magnification images. **Figure S6.** Images of Col I and Col II staining of specimens at 6 weeks of implantation. The scale bars are 500 μm in low magnification images and 200 μm in high magnification images.

## Data Availability

The raw data required to reproduce these findings are available on reasonable request from the corresponding author (Z.X.).

## Abbreviations

hUSCs: Human urine-derived stem cells
hBMSCs: Human bone marrow mesenchymal stem cells
ACM: Acellular cartilage extracellular matrix
hADSCs: Human adipose-derived stem cells
hPDB-MSCs: Human placenta decidua basalis-derived mesenchymal stem cells
iPSCs: Induced pluripotent stem cells
Col II: Type II collagen
GAGs: Glucosaminoglycans
PBS: Phosphate-buffered saline
DMEM-HG: Dulbecco’s modified Eagle’s medium-high glucose
EDTA: Ethylenediaminetetraacetic acid
K-SFM: Keratinocyte serum-free medium
EGF: Epidermal growth factor
BPE: Bovine pituitary extract
CCK-8: Cell Counting Kit-8
OD: Optical density
2D: Two-dimensional
3D: Three-dimensional
ALP: Alkaline phosphatase
ARS: Alizarin red S
ORO: Oil red O
qRT-PCR: Quantitative reverse transcription polymerase chain reaction
Agg: Aggrecan
Col I: Collagen I
PFA: Paraformaldehyde
H&E: Hematoxylin and eosin
EDC: 1-Ethyl-3-(3-dimethylaminopropyl) carbodiimide hydrochloride
NHS: *N*-hydroxy succinimide
SEM: Scanning electron microscopy
Masson: Masson’s trichrome
dsDNA: Double-standard DNA
MRI: Magnetic resonance imaging
μCT: Micro-computed tomography
BV: Bone volume
TV: Tissue volume
IL-1: Inflammatory factor interleukin-1
TNF-α: Tumor necrosis factor-a
ELISA: Enzyme-linked immunosorbent assay
SDs: Standard deviations
P0: Primary passage
ECM: Extracellular matrix
