# Correction: Comparison of chondrogenesis-related biological behaviors between human urine-derived stem cells and human bone marrow mesenchymal stem cells from the same individual

**DOI:** 10.1186/s13287-022-03193-4

**Published:** 2022-11-09

**Authors:** Jiachen Sun, Fei Xing, Min Zou, Min Gong, Lang Li, Zhou Xiang

**Affiliations:** 1 Department of Orthopedics, West China Hospital, Sichuan University, Guoxue Lane 37, Chengdu, Sichuan 610041 People’s Republic of China; 2 Department of Orthopedics, NO. 1 People’s Hospital of Chengdu, Chengdu, Sichuan 610016 People’s Republic of China; 3 Department of Orthopedics, Hospital of Chengdu University of Traditional Chinese Medicine, Chengdu, Sichuan 610075 People’s Republic of China; 4 Department of Orthopaedics, Hospital of Chengdu Office of People’s Government of Tibetan Autonomous Region, NO.20 Ximianqiao Cross Street, Chengdu, Sichuan 610041 People’s Republic of China

## Correction to: Stem Cell Research & Therapy (2021) 12:366 https://doi.org/10.1186/s13287-021-02370-1

In the article titled “Comparison of Chondrogenesis-Related Biological Behaviors Between Human Urine-Derived Stem Cells and Human Bone Marrow Mesenchymal Stem Cells from the Same Individual” [1], we notice these following errors:

(i) The image in Fig. 2A of hUSCs at P7 was overlaid with the image of hUSCs at P5 when we rearranged the image layout.
(ii) The image in the first row in Fig. 5C is not the image of hUSCs at P3 growing on an acellular cartilage matrix scaffold. We made an error while renaming the image during file transfer.

The corrected figures are shown herein.

**Fig. 2:**
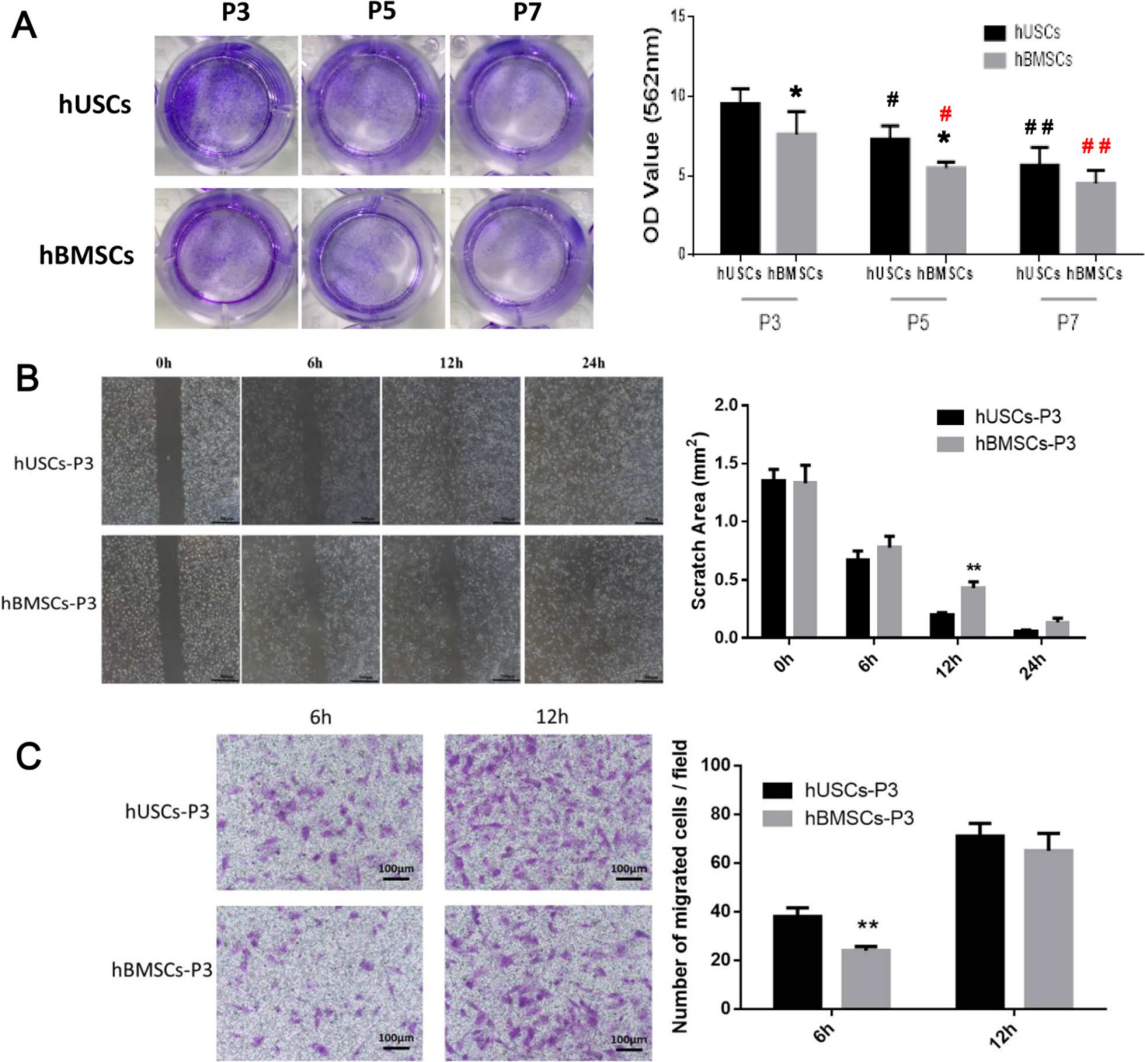
**a** Colony formation analysis of hUSCs and hBMSCs at P3, P5, and P7. **b** Scratch wound closure assay of hUSCs and hBMSCs at P3. Scale bar = 500 μm. **c** Transwell migration assay of hUSCs and hBMSCs at P3. Scale bar = 100 μm. *p < 0.05 and **p < 0.01, hBMSCs compared to hUSCs at the same passage; #*p* < 0.05 and ##*p* < 0.01, hUSCs compared to hUSCs at P3; #*p* < 0.05 and ##*p* < 0.01 in red, hBMSCs compared to hBMSCs at P3.

**Fig. 5:**
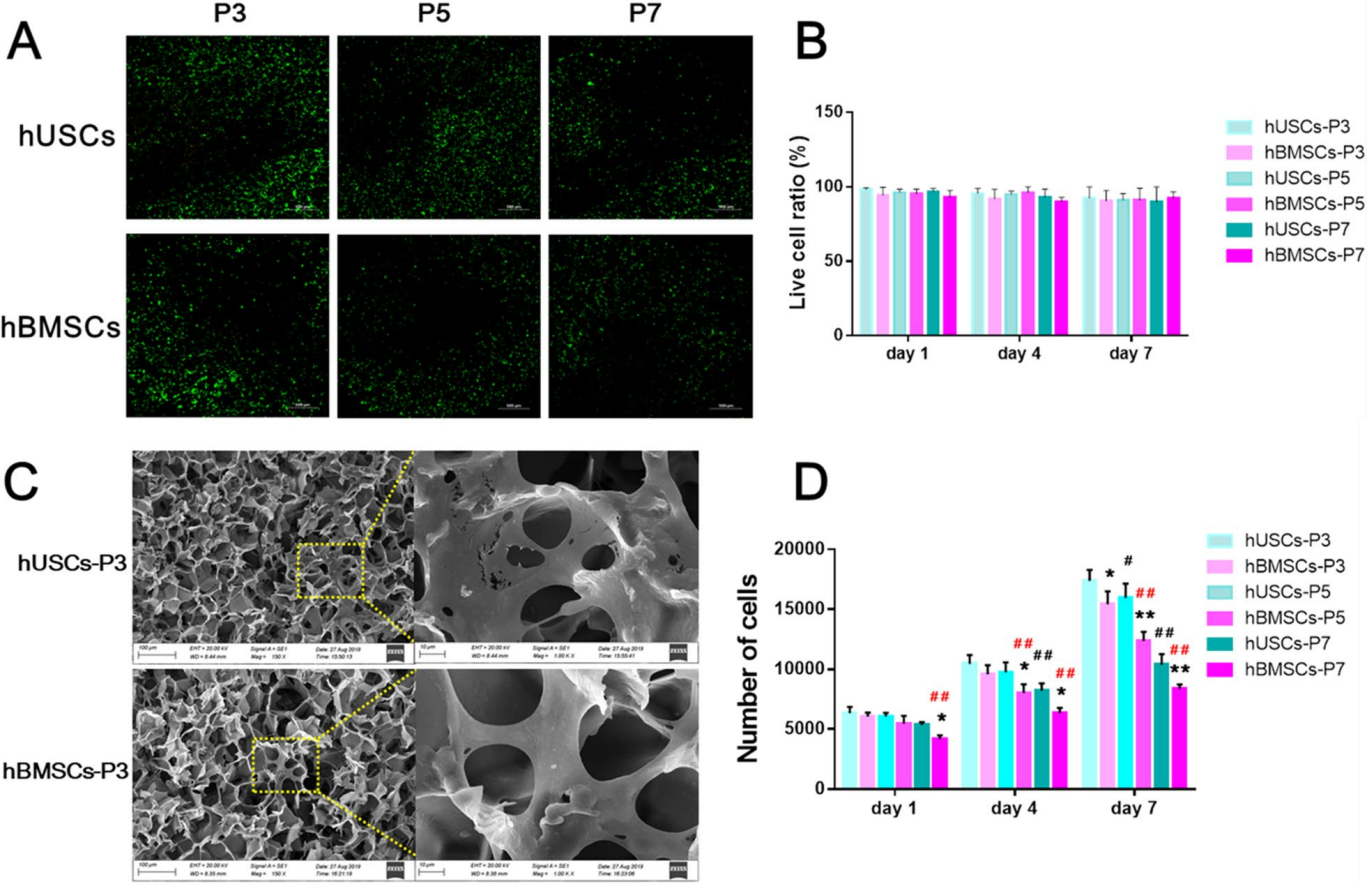
**a** Live (green) and dead (red) cells of hUSCs and hBMSCs cultured on scaffolds after 3 days, via live-dead staining. **b** Live cell ratio in live-dead staining results. **c** SEM images of cell adhesion on scaffolds after seeding for 3 days. The scale bars are 100 μm in low magnification images and 10 μm in high magnification images. **d** Cell proliferation of cells cultured on scaffolds after 1, 4, and 7 days, via the detection of DNA content. *p < 0.05 and **p < 0.01, hBMSCs compared to hUSCs at the same passage; #*p* < 0.05 and ##*p* < 0.01, hUSCs compared to hUSCs at P3; #*p* < 0.05 and ##*p* < 0.01 in red, hBMSCs compared to hBMSCs at P3.
