# Supplementary material for: Comparison of chondrogenesis-related biological behaviors between human urine-derived stem cells and human bone marrow mesenchymal stem cells from the same individual
Source: Stem Cell Res Ther. 2021 Jun 28;12:366. doi: 10.1186/s13287-021-02370-1 (PMC8240221; doi:10.1186/s13287-021-02370-1)
Supplement: Supplementary file 1 — Additional file 1: Figure S1. Osteogenic and adipogenic induction results of hUSCs and hBMSCs at P3, P5, and P7. (A) ALP staining and normalized ALP activity detected at 14 days of osteogenic induction. Scale bar = 200 μm. (B) ARS staining and normalized Alizarin red intensity at 21 days of osteogenic induction. Scale bar = 200 μm. (C) ORO staining and normalized ORO intensity at 14 days of adipogenic induction. Scale bar = 100 μm. *p < 0.05 and **p < 0.01, hBMSCs compared to hUSCs at the same passage; #p < 0.05 and ##p < 0.01, hUSCs compared to hUSCs at P3; #p < 0.05 and ##p < 0.01 in red, hBMSCs compared to hBMSCs at P3. Figure S2. (A) Biochemical assay of the unacellular cartilage slices and ACM– scaffolds, including the contents of DNA, GAG, and total collagen. (B) SEM images of unacellular cartilage slices, ACM–, and ACM+. The scale bar is 100 μm (100 ×) or 20 μm (600 ×). (C) The pore size, porosity, and swelling rate of ACM– and ACM+. Statistically significant differences are indicated with *p < 0.05 and **p < 0.01. Figure S3. (A) Histological staining results of ACM– and ACM+ scaffolds, including H&E, Masson’s, safranin O, and toluidine blue. The scale bar is 500 μm (40 ×) or 200 μm (100 ×). (B) Contact angle detection of ACM– and ACM+. (C) Compressive elastic modulus of ACM– and ACM+. Statistically significant differences are indicated with *p < 0.05 and **p < 0.01. Figure S4. (A) H&E staining images of the synovium in the joint at 6 weeks; scale bar = 50 μm. (B) IL-1 and TNF-α contents in synovial fluid at 6 weeks. Statistically significant differences are indicated with *p < 0.05 and **p < 0.01. (C) H&E staining images of livers, lungs, and kidneys at 6 weeks; scale bar = 500 μm. Figure S5. Images of H&E and Masson staining of specimens at 6 weeks of implantation. The scale bars are 500 μm in low magnification images and 200 μm in high magnification images. Figure S6. Images of Col I and Col II staining of specimens at 6 weeks of implantation. The scale ba [file 13287_2021_2370_MOESM1_ESM.docx]

**Comparison of** **Chondrogenesis-Related Biological Behaviors Between** **Human Urine-Derived Stem Cells and** **Human Bone Marrow Mesenchymal Stem Cells from the Same Individual**

Jiachen Sun^[1, †]^, Lang Li^[1, 2, †]^, Min Zou^[3]^, Min Gong^[4]^, and Zhou Xiang* ^[1]^

[1] *Dr. J. Sun, Dr. L. Li, and Prof. Dr. Z. Xiang*
Department of Orthopedics, West China Hospital, Sichuan University, Chengdu, Sichuan 610041, P. R. China

[2] *Dr. L. Li*
Department of Orthopaedics, Hospital of Chengdu Office of People’s Government of Tibetan Autonomous Region, Chengdu, Sichuan 610041, P. R. China

[3] *Dr. M. Zou*Department of Orthopedics, NO. 1 People’s hospital of chengdu, Chengdu, Sichuan 610016, P. R. China

[4] *Dr. M. Gong*Department of Orthopedics, Hospital of Chengdu University of Traditional Chinese Medicine, Chengdu, Sichuan 610075, P. R. China

[^†^] These two authors contributed equally to this work.

* Corresponding author:

*Prof. Dr. Z. Xiang,* Department of Orthopedics, West China Hospital, Sichuan University, Guoxue Lane 37, Chengdu 610041, Sichuan Province, P. R. China.

Email: [xiangzhou15@hotmail.com](mailto:xiangzhou15@hotmail.com) (Z. Xiang).

**
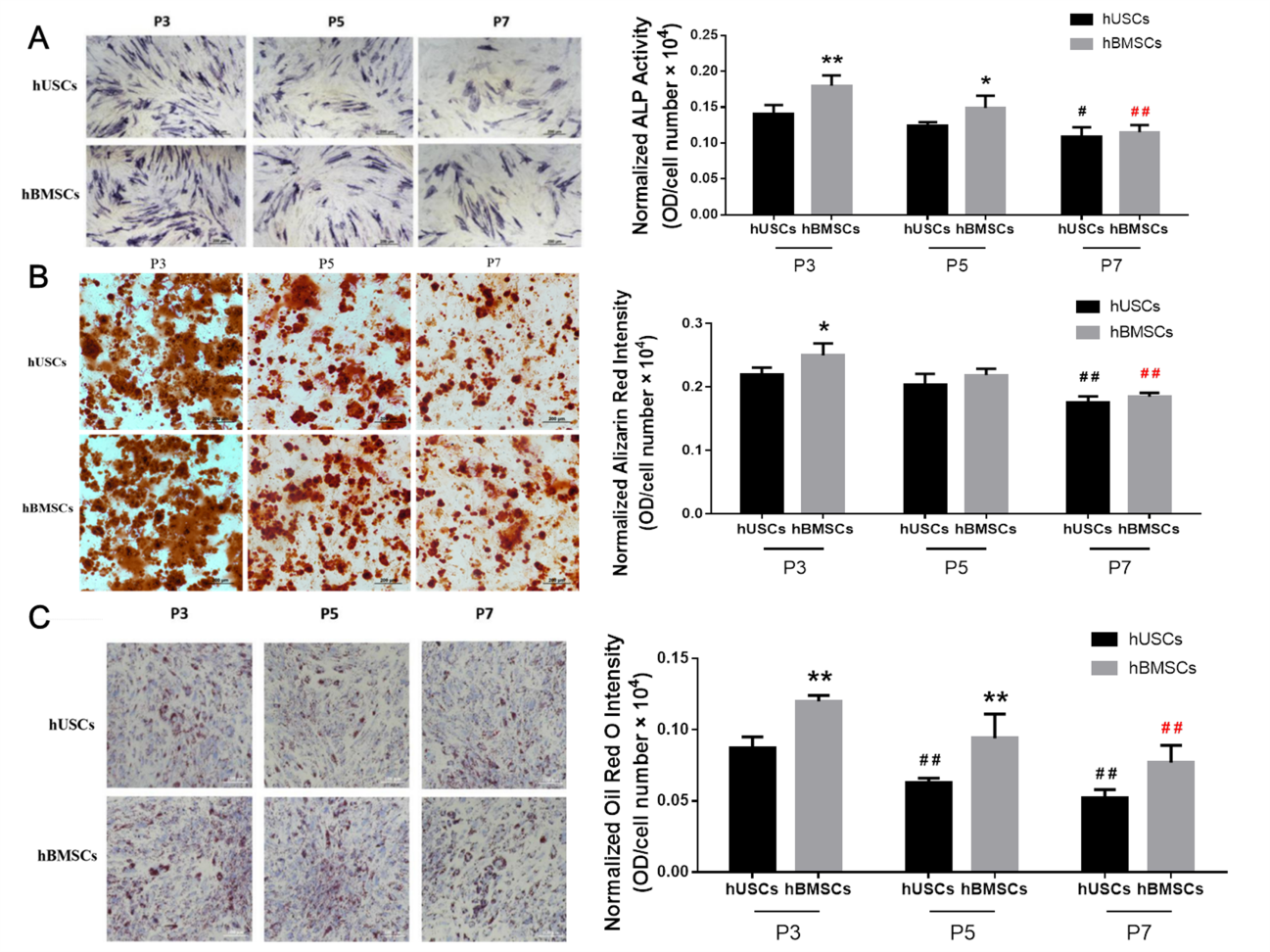
**

**Figure S1.** Osteogenic and adipogenic induction results of hUSCs and hBMSCs at P3, P5, and P7. (A) ALP staining and normalized ALP activity detected at 14 days of osteogenic induction. Scale bar = 200 μm. (B) ARS staining and normalized Alizarin red intensity at 21 days of osteogenic induction. Scale bar = 200 μm. (C) ORO staining and normalized ORO intensity at 14 days of adipogenic induction. Scale bar = 100 μm. *p < 0.05 and **p < 0.01, hBMSCs compared to hUSCs at the same passage; ^#^p < 0.05 and ^##^p < 0.01, hUSCs compared to hUSCs at P3; ^#^p < 0.05 and ^##^p < 0.01 in red, hBMSCs compared to hBMSCs at P3.


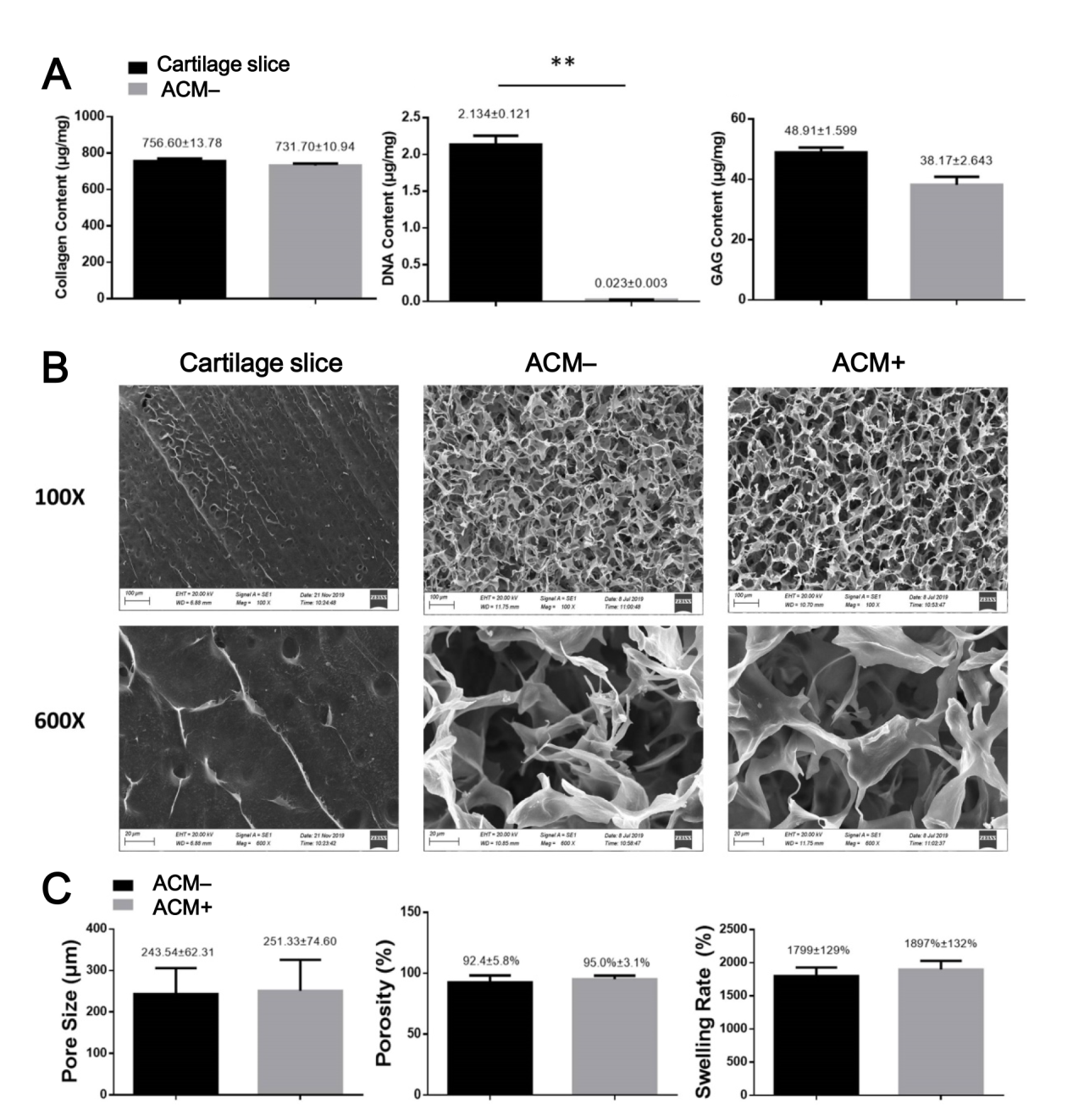


**Figure S2.** (A) Biochemical assay of the unacellular cartilage slices and ACM– scaffolds, including the contents of DNA, GAG, and total collagen. (B) SEM images of unacellular cartilage slices, ACM–, and ACM+. The scale bar is 100 μm (100 ×) or 20 μm (600 ×). (C) The pore size, porosity, and swelling rate of ACM– and ACM+. Statistically significant differences are indicated with *p < 0.05 and **p < 0.01.


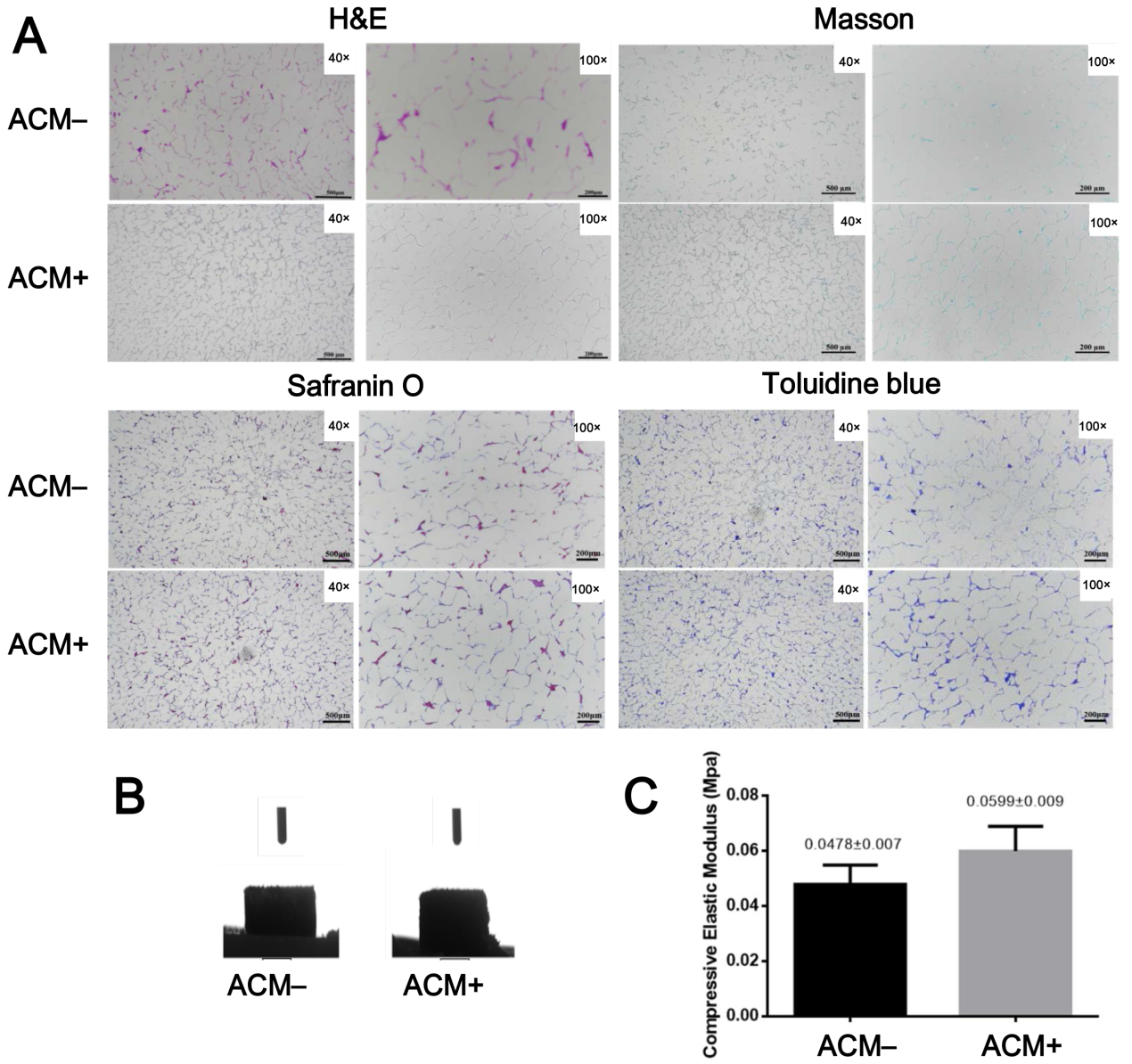


**Figure S3.** (A) Histological staining results of ACM– and ACM+ scaffolds, including H&E, Masson’s, safranin O, and toluidine blue. The scale bar is 500 μm (40 ×) or 200 μm (100 ×). (B) Contact angle detection of ACM– and ACM+. (C) Compressive elastic modulus of ACM– and ACM+. Statistically significant differences are indicated with *p < 0.05 and **p < 0.01.


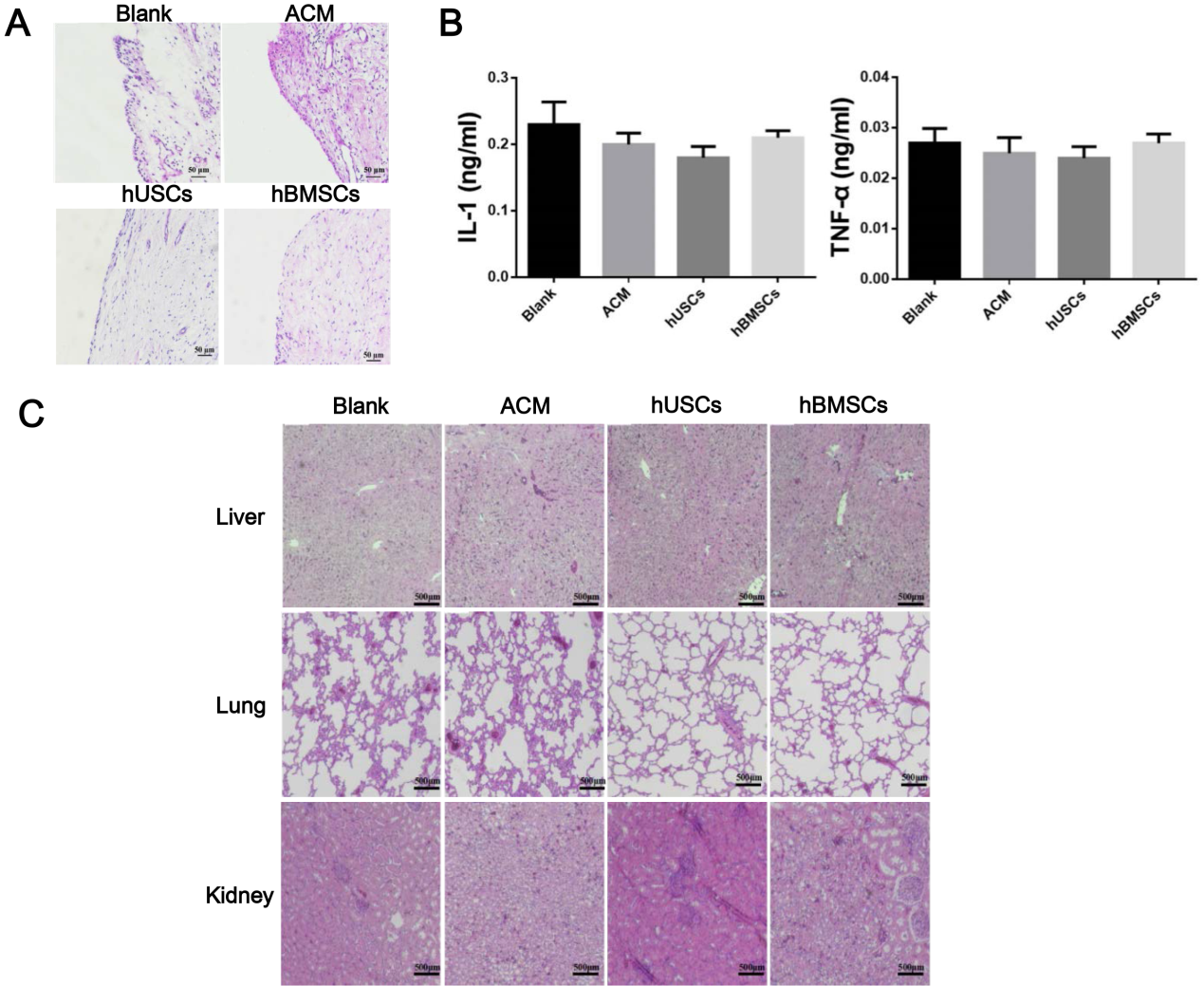
**Figure S4.** (A) H&E staining images of the synovium in the joint at 6 weeks; scale bar = 50 µm. (B) IL-1 and TNF-α contents in synovial fluid at 6 weeks. Statistically significant differences are indicated with *p < 0.05 and **p < 0.01. (C) H&E staining images of livers, lungs, and kidneys at 6 weeks; scale bar = 500 µm.


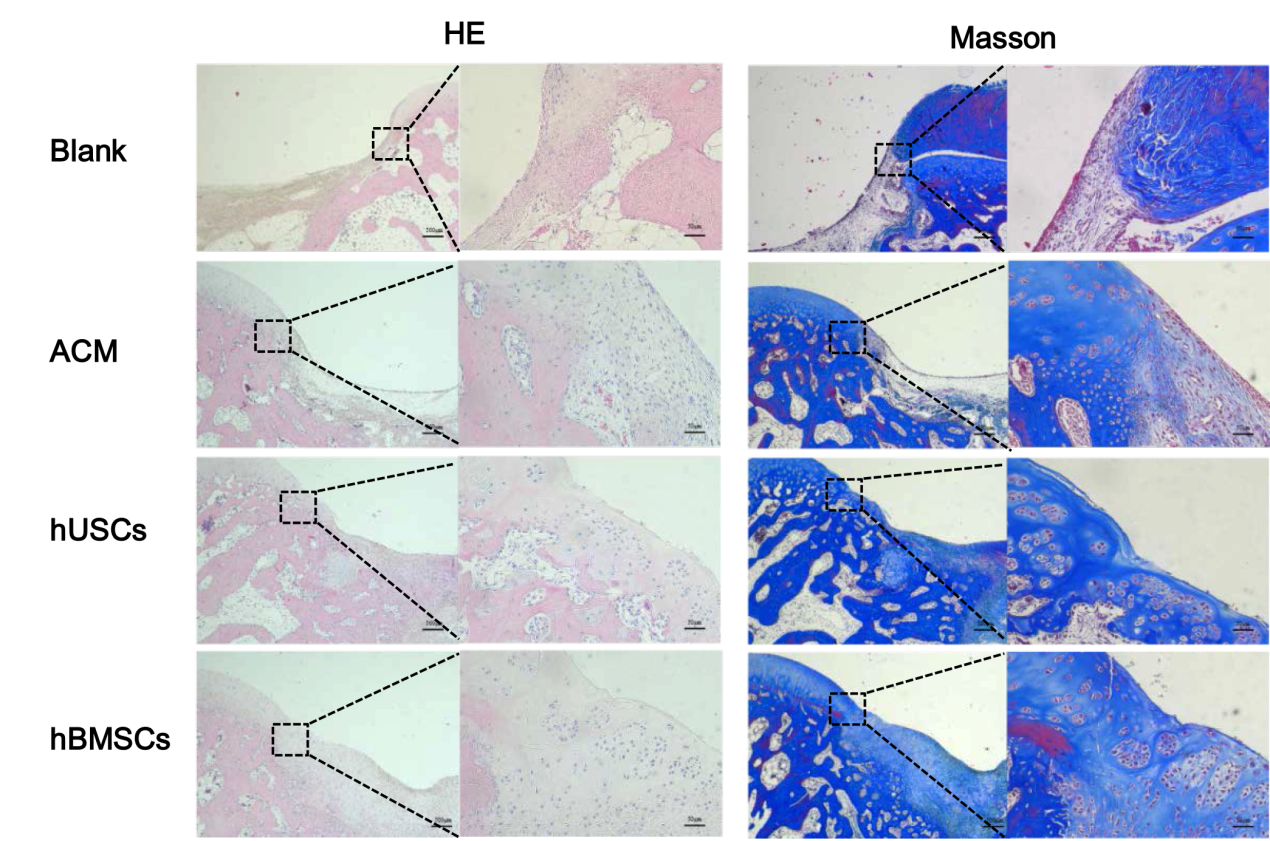


**Figure S5.** Images of H&E and Masson staining of specimens at 6 weeks of implantation. The scale bars are 500 μm in low magnification images and 200 μm in high magnification images.


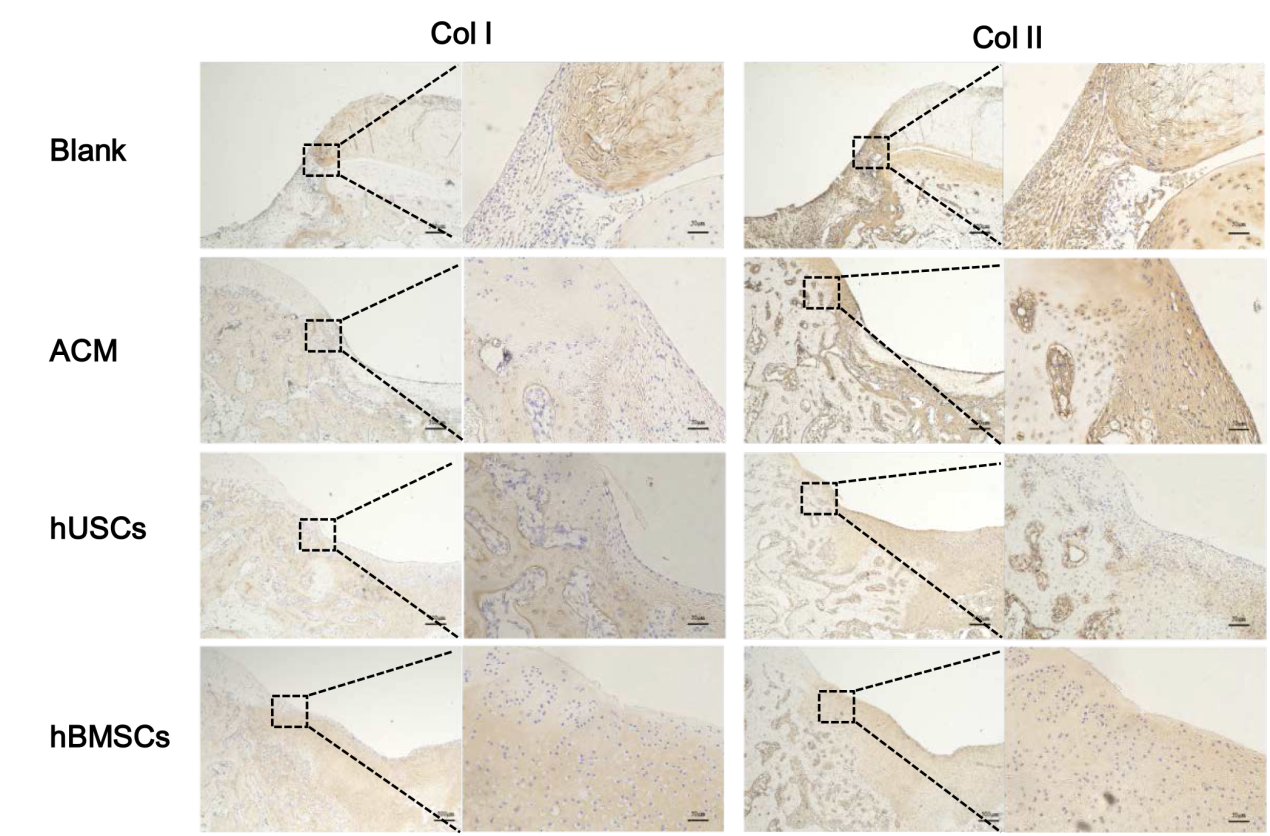


**Figure S6.** Images of Col I and Col II staining of specimens at 6 weeks of implantation. The scale bars are 500 μm in low magnification images and 200 μm in high magnification images.
